# ACA-Net: adaptive context-aware network for basketball action recognition

**DOI:** 10.3389/fnbot.2024.1471327

**Published:** 2024-09-25

**Authors:** Yaolei Zhang, Fei Zhang, Yuanli Zhou, Xiao Xu

**Affiliations:** ^1^China Basketball College, Beijing Sport University, Beijing, China; ^2^College of Physical Education, Hangzhou Normal University, Hangzhou, Zhejiang, China; ^3^Radar Non-Commissioned Officers' School, Air Force Early Warning Academy, Wuhan, Hubei, China; ^4^College of Physical Education, Dalian University, Dalian, Liaoning, China

**Keywords:** basketball, action recognition, adaptive context-awareness, long short-term information, space-channel information interaction

## Abstract

The advancements in intelligent action recognition can be instrumental in developing autonomous robotic systems capable of analyzing complex human activities in real-time, contributing to the growing field of robotics that operates in dynamic environments. The precise recognition of basketball players' actions using artificial intelligence technology can provide valuable assistance and guidance to athletes, coaches, and analysts, and can help referees make fairer decisions during games. However, unlike action recognition in simpler scenarios, the background in basketball is similar and complex, the differences between various actions are subtle, and lighting conditions are inconsistent, making action recognition in basketball a challenging task. To address this problem, an Adaptive Context-Aware Network (ACA-Net) for basketball player action recognition is proposed in this paper. It contains a Long Short-term Adaptive (LSTA) module and a Triplet Spatial-Channel Interaction (TSCI) module to extract effective features at the temporal, spatial, and channel levels. The LSTA module adaptively learns global and local temporal features of the video. The TSCI module enhances the feature representation by learning the interaction features between space and channels. We conducted extensive experiments on the popular basketball action recognition datasets SpaceJam and Basketball-51. The results show that ACA-Net outperforms the current mainstream methods, achieving 89.26% and 92.05% in terms of classification accuracy on the two datasets, respectively. ACA-Net's adaptable architecture also holds potential for real-world applications in autonomous robotics, where accurate recognition of complex human actions in unstructured environments is crucial for tasks such as automated game analysis, player performance evaluation, and enhanced interactive broadcasting experiences.

## 1 Introduction

In recent years, the integration of neural network models in autonomous systems has revolutionized various fields, including robotics, and computer vision, enabling machines to perform complex tasks in dynamic environments (Gan et al., 2024; Saleem et al., 2023; Babaee Khobdeh et al., 2021). One of the most promising applications of these advancements is in sports analytics, where intelligent systems can autonomously monitor, analyze, and interpret athlete movements. These systems not only provide precise technical guidance to athletes, coaches, and analysts, but also assist referees in making fairer decisions during matches, enhancing the fairness and accuracy of the game. The growing field of autonomous sports analysis leverages machine learning and neural network models to optimize training, improve competition outcomes, and enhance the overall fan experience. Among these sports, basketball stands out as one of the most popular and globally influential. Accurate recognition of basketball players' movements during training and games is critical for coaches, enabling them to design targeted training plans and strategies based on objective data rather than subjective observations. Traditionally, coaches have relied on their experience and time-intensive manual analysis to evaluate player performance, leading to inefficiencies and potential inaccuracies (Wei et al., 2016). Leveraging computer vision technology for autonomous recognition of player movements addresses these challenges, offering a foundation for advanced applications such as automatic detection of key game events, intelligent analysis of basketball tactics, and automatic generation of game highlights. These innovations, powered by neural network models, can significantly enhance both the technical level of athletes and the overall spectacle of the game (Li and Zhang, 2019). Additionally, these advancements in action recognition hold great potential for broader applications in autonomous robotic systems, where accurate, real-time recognition of human actions is essential for intelligent decision-making and interaction in complex, unstructured environments (Jain et al., 2023; Wang et al., 2022).

Basketball action recognition techniques can be divided into two main routes: the first is recognition using inertial sensors and the second is recognition using the feature extraction from video or image. Li and Gu (2021) used inertial sensors to measure the acceleration and angular velocity of the arm to help recognize basketball actions. Gun (2021) proposed the integration of a Field Programmable Gate Array (FPGA) into a network of two data streams to find the optimal region for recognizing basketball actions. These methods require athletes to be equipped with specific sensors that collect data and send it to a processing device for action analysis, but is not suitable for general use due to its equipment dependency. Liu and Wang (2023) used wearable sensors to capture user motion data, then optimize the model to analyse and recognize user behavior through SVM algorithm. Liu and Liu (2023) leveraged the wearable device to collect three-axis acceleration data and three-axis angular velocity data of the basketball player for action recognition. Jiang and Zhang (2023) proposed a scientific structure for classifying motion postures is proposed, which leads to the establishment of a data information acquisition module based on inertial sensors. The convolutional neural network is then improved using principal component analysis, and finally the improved algorithm is applied to identify basketball poses.

The methods based on feature extraction use video or image captured by a camera, from which hidden features are extracted and then recognized using a neural network classifier. This type of method is currently the most widely used in action recognition. Researchers are beginning to extend 2D convolution to 3D convolution to capture features in the time dimension, e.g., C3D (Tran et al., 2015), TSN (Wang et al., 2016), I3D (Carreira and Zisserman, 2017), P3D (Qiu et al., 2017), Non-local (Wang et al., 2018), R(2+1)D (Tran et al., 2018), R3D (Hara et al., 2018), and SlowFast (Feichtenhofer et al., 2019). DeepVideo (Karpathy et al., 2014) is one of the first attempts to use convolutional neural networks for video. C3D (Convolutional 3D) is currently the most popular neural network for extracting video features, as it is capable of extracting features in the temporal dimension in addition to the spatial dimension compared to 2D convolution (Fan et al., 2016; Li et al., 2017; Xu et al., 2017; de Melo et al., 2019; Yang et al., 2021). Donahue et al. (2015) explored the most suitable C3D convolution kernel size for action recognition through experimental studies. In order to further improve the representation and generalization ability, researchers have successively proposed 3D residual convolutional network (Tran et al., 2017) and pseudo-3D residual network (Qiu et al., 2017). Wu et al. (2020) proposed a dual-stream 3D convolutional network that uses optical flow information to obtain global and local action features. Gu et al. (2020) introduced the Navigator-Teacher-Scrutiniser Network into the dual-stream network with the aim of focusing on the most informative regions for fine-grained action recognition. In recent years, the most significant attention-based Transformer model has been proposed by Vaswani et al. (2017). Using Transformer's multi-head self-attention layer, it is possible to compute a representation of a sequence by aligning words in the sequence with other words in the sequence. It performs better in terms of representation and uses less computational resources than convolutional and recurrent operations.The success of the Transformer model inspired the computer vision community to test it on video tasks, such as ViVIT (Arnab et al., 2021), Video-Swin (Liu et al., 2022), and TimesFormer (Bertasius et al., 2021). Moreover, Peng et al. (2020) proposed a novel spatial-temporal GCN (ST-GCN) architecture for 3D skeleton-based action recognition, which is able to better model the latent anatomy of the structure data. Huang et al. (2023) utilized ensemble models based on hypergraph-convolution Transformer for the MiG classification from human skeleton data. The method effectively extracts subtle dynamic features from different gestures by enhancing the attention mechanism and multi-model fusion techniques.

Although deep learning-based algorithms are capable of recognizing basketball actions, they face some specific challenges such as dealing with highly similar complex backgrounds, subtle action differences within frames, and inconsistent lighting conditions. These factors significantly impact the accuracy and robustness of action recognition in basketball videos. To address these issues, we propose an Adaptive Context-Aware Network (ACA-Net) deep neural network for basketball action recognition to solve the problem that the current popular methods unable to extract sufficient features and fails to capture long term temporal information, resulting in low accuracy in recognizing basketball player's actions. The main contributions of this paper are as follows:

1. We propose an adaptive context-aware network (ACA-Net) for basketball action recognition. By aggregating long short-term temporal features with spatial-channel features, the network can effectively recognize basketball actions guided by video contextual information.
2. We propose a long short-term adaptive learning mechanism. To address the problem of difficulty in determining similar basketball actions from short-term temporal information, it first filters features through a convolutional gating mechanism, then generates a dynamic convolutional kernel for video adaptation to aggregate temporal information and capture long-term dependencies in videos, and finally generates importance weights from short-term temporal information to enhance temporal feature representation. Thus, it can adaptively integrate contextual information from different time scales to enhance the model's ability to capture long-term temporal information.
3. We propose a triplet spatial-channel integration strategy, which performs cross-dimensional interactions between spatial and channel dimensions, and spatial intrinsic dimensions on each of the three branches, which complementarily improves the representation of the features in the network. Thus it can efficiently extract more discriminative feature representations from basketball videos with a large number of similar backgrounds.

The rest of the paper is organized as follows. Section 2 introduces the adaptive context-aware network, the long short-term adaptive learning mechanism, and the triplet spatial-channel interaction strategy. Section 3 introduces the datasets, evaluation metrics, and the experiments to verify the effectiveness of the methods in this paper. Sections 4, 5 introduce the discussion and conclusions of this study.

## 2 Methodology

### 2.1 The overview of adaptive context-aware network

As we discussed in Section 1, basketball videos pose difficulties in modeling the long-term dependency of the videos as well as discriminative feature representations due to the complexity of the background and the similarity of the actions. Therefore, we aim to address the above problems by introducing a long short-term adaptive (LSTA) module and a triplet spatial-channel interaction module. The two proposed modules can be easily integrated into existing 2D CNNs, such as ResNet (He et al., 2016), to form a network architecture that can efficiently process basketball videos. We will give an overview of ACA-Net and then describe the technical details of the proposed modules.

**Figure 1:**
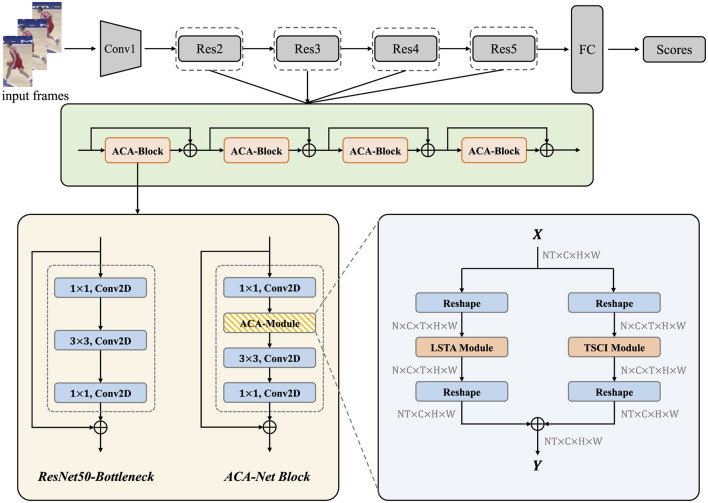
The overall architecture of ACA-Net. The vanilla Bottlenecks in ResNet50 are replaced with ACA-Net Blocks to instantiate ACA-Net. The whole workflow of ACA-Module in the lower right shows how it works. We have noted the shape of tensor after each step. ⊕ denotes element-wise addition.

As shown in the Figure 1, we adopt ResNet50 as the backbone and insert the Adaptive Context-Aware Module (ACA-Module) after the first 1 × 1 convolution of each bottleneck of the layers in ResNet50. We call the improved bottleneck the ACA-Block. Each ACA-module in ACA-Block contains an LSTA module and a TSCI module to extract features from different aspects of the video. The LSTA module strengthens the model's performance in extracting the long-term dependency information of the video, and the TSCI module enhances the model's ability to extract discriminative features through the cross-dimensional interactions between the space and the channels. Next we will introduce the technical details of the LSTA module and the TSCI module.

### 2.2 LSTA module

**Figure 2:**
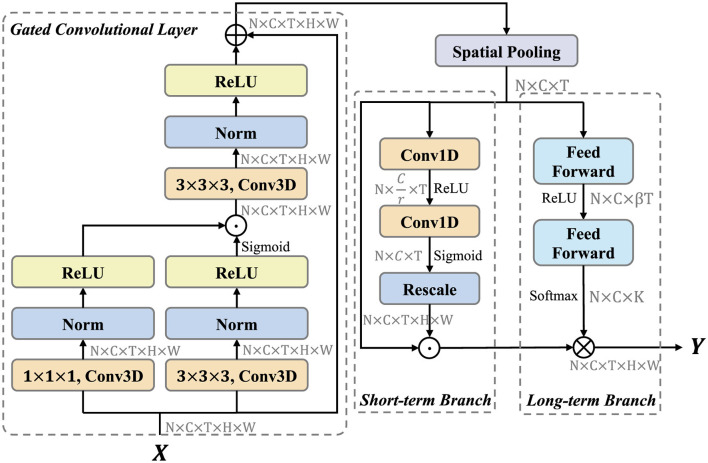
LSTA module architecture. LSTA module contains three parts: Gated Convolutioal Layer, Short-term branch, and Long-term Branch. We have noted the shape of tensor after each step. ⊕ denotes element-wise addition. ⊗ represents convolution operation. ⊙ is element-wise multiplication.

The LSTA module is designed to adaptively learn the long and short term information of basketball videos. As shown in the Figure 2, the LSTA module consists of three main parts, which are gated convolutional layer, short-term branch and long-term branch.

Formally, let *X*∈ℝ^*C*×*T*×*H*×*W*^ denotes the input feature map, where *C* indicates the number of channels, *T* represents the temporal dimension, and *H, W* are the spatial dimensions. For convenience, the dimension of batch size *N* is ignored here and in the following descriptions of shapes. First, we leverage gated convolutional layer to enhance the feature representation. The feature map *X*∈ℝ^*C*×*T*×*H*×*W*^ are fed into two convolution sublayers, which contains a convolution, a instance norm (Ulyanov et al., 2016), and a ReLU layer (Glorot et al., 2011). The convolution kernel size is 3 × 3 × 3 and 1 × 1 × 1, respectively. These two features are then multiplied pixel by pixel to control the information transfer, similar to the gate mechanism (Liu et al., 2021). This process can be formalized as


(1) $$\begin{array}{l} G=X⊕Conv3D^{3\times 3\times 3}\left(Conv3D^{3\times 3\times 3}\left(X\right)⊙Conv3D^{1\times 1\times 1}\left(X\right)\right) \end{array}$$

where *X* denotes the input feature map and *C* denotes the convolution block. Then, the output *G*^*C*×*T*×*H*×*W*^ is fed into short-term and long-term branch. For efficiency, these two branches only focuses on temporal modeling. Therefore, We first use global spatial average pooling to compress the feature map as follows:


(2) $$\begin{array}{l} P=\frac{1}{H\times W}\underset{i,j}{\sum }G_{c,t,j,i}, \end{array}$$

where *c, t, i, j* denotes the index of channel, time, height and width dimensions. *P*^*C*×*T*^ aggregates the spatial information of *G*^*C*×*T*×*H*×*W*^. The aim of short-term branch is to extract the short-term information and generate the location-related importance weights. To control the model complexity, the first Conv1D operation reduce the number of channels form *C* to $\frac{C}{r}$. The second Conv1D operation recover the number of channels to *C*, and yields an importance weights *V*^*C*×*T*^ through the sigmoid function. Above process can be formulated as follows:


(3) $$\begin{array}{l} V=F_{rescale}\left(\sigma \left(Conv1D\left(\delta \left(Conv1D\left(P,\frac{C}{r}\right)\right),C\right)\right)\right), \end{array}$$

where *P* is the feature map form global spatial average pooling, δ and σ denote the ReLU and Sigmoid activation function, respectively. *F*_*rescale*_ rescales ${\hat{V}}^{C\times T}$ to *V*^*C*×*T*×*H*×*W*^ by replicating in spatial dimension. Finally, the feature map *P* is multiplied element-wise with the importance weights *V*.


(4) $$\begin{array}{l} Z=P⊙V \end{array}$$

The Long-term Branch is mainly responsible for long-range temporal modeling and capturing long-range dependencies in videos. We generate dynamic temporal aggregation convolution kernels for the diversity of video temporal information and aggregate them in a convolutional manner. In order to simplify the generation of adaptive convolutional kernels and maintain a high inference efficiency, we adopt a channel-by-channel temporal sequence convolutional kernel generation method. Based on this idea, we expect that the generated adaptive convolution kernel only considers modeling temporal relationships, and the generation process of the convolution kernel is summarized as follows:


(5) $$\Theta_{c}=softmax(ℱ(W_{2},\delta (ℱ(W_{1},P))))$$

where $\Theta_{c}\in {\mathbb{R}}^{K}$ is the adaptive convolution kernel for the *c*^*th*^ channel, *K* is the kernel size, δ denotes the ReLU activation function, ℱ is the fully connected feed forward layer, and **W**_1_ and **W**_2_ are the learnable parameters. Next, the generated adaptive kernel Θ = {Θ_1_, Θ_2_, …, Θ_*C*_} learns the temporal structure information between video frames in the convolution manner for temporal adaptive aggregation:


(6) $$\begin{array}{l} Y=\Theta ⊗Z=\overset{K}{\underset{k}{\sum }}\Theta_{c,k}\cdot Z_{c,t+k,j,i} \end{array}$$

where ⊗ denotes channel-by-channel temporal convolution, *Z* is the feature map output from short-term branch.

In summary, LSTA module is capable of generating adaptive convolution kernels to aggregate short-term and long-term temporal features of the basketball video while maintaining computational efficiency.

### 2.3 TSCI module

**Figure 3:**
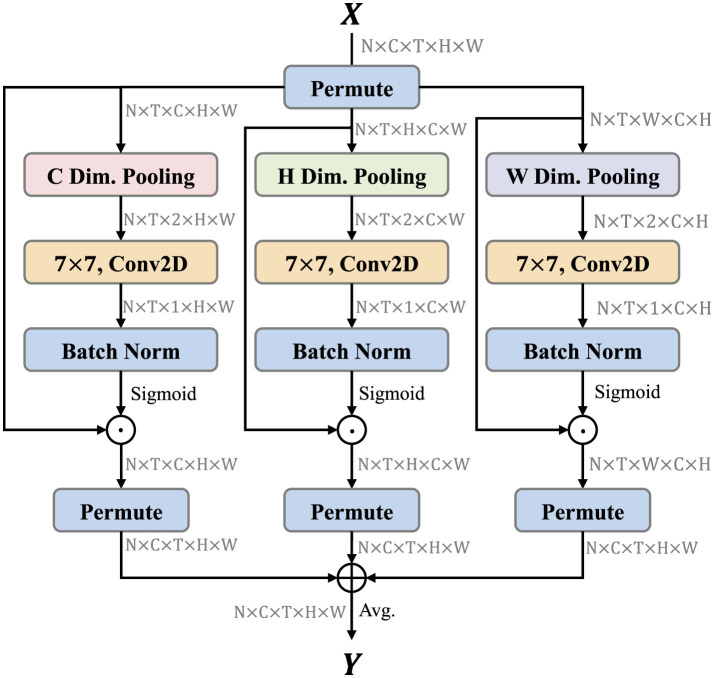
TSCI module architecture. TSCI module contains three branches to focus different aspects of information. We have noted the shape of tensor after each step. ⊕ denotes element-wise addition, and ⊙ is element-wise multiplication.

Triplet attention (Misra et al., 2021) is a light-weight but effective attention mechanisms for computer vision tasks, such as image classification, which computes attention weights by capturing cross-dimension interaction using a three-branch structure. Inspired by this idea, we extend triplet attention to video and propose the TSCI module. The TSCI module builds inter-dimensional dependencies by the rotation operation followed by residual transformations and encodes inter-channel and spatial information with negligible computational overhead. The architecture of TSCI module is shown as Figure 3. The objective of the TSCI module is to enhance the model's ability to extract discriminative features through cross-dimensional interactions between three different dimensions, i.e., (*C, H*), (*C, W*), and (*H, W*), respectively.

Formally, let *X*∈ℝ^*C*×*T*×*H*×*W*^ denotes the input feature map, we first permute the spatial and channel dimensions. $X_{C}\in {\mathbb{R}}^{T\times C\times H\times W}$ is obtained in the left branch after dimensional permuting. The global average pooling and global max pooling are performed along the direction *C* to obtain the feature map $X_{C}^{\prime }\in {\mathbb{R}}^{T\times 2\times H\times W}$, which can establish the information interaction between *H* and *W*. Similarly, $X_{H}\in {\mathbb{R}}^{T\times H\times C\times W}$ is obtained in the middle branch after dimensional permuting, and global average pooling and global max pooling are performed along the direction *H* to obtain the feature map $X_{H}^{\prime }\in {\mathbb{R}}^{T\times 2\times C\times W}$, which can establish the information interaction between *C* and *W*. $X_{W}\in {\mathbb{R}}^{T\times W\times C\times H}$ is obtained in the right branch after dimensional permuting, and global average pooling and global max pooling are performed along the direction *W* to obtain the feature map $X_{W}^{\prime }\in {\mathbb{R}}^{T\times 2\times C\times H}$, which can establish the information interaction between *C* and *H*. Above pooling process can be formulated as follows:


(7) $$\text{Dimensional Pooling}(X)=\text{Concat}[{\text{Avgpool}}_{\text{3d}}(X),{\text{Maxpool}}_{\text{3d}}(X)]$$

Secondly, we leverage the features output from the above process to generate the attention map. Three two-dimensional convolution layers are performed for features $X_{C}^{\prime }$, $X_{H}^{\prime }$, and $X_{W}^{\prime }$, respectively. The attention map *w*_*C*_, *w*_*H*_, and *w*_*W*_ are obtained through the batch normalization layer (Ioffe and Szegedy, 2015) and sigmoid activation function. Then, they are multiplied element-wise by the *X*_*C*_, *X*_*H*_, and *X*_*W*_ of the corresponding branch to pay more attention to the region of interest.

Finally, the output feature maps of the three branches are averaged to achieve the aggregation of features. The whole process can be represented as follows:


(8) $$\begin{array}{l} Y=\mathrm{Avg}(\bar{X_{C}⊙\sigma (\mathrm{Conv}2D(X_{C}^{\prime }))}+\bar{X_{H}⊙\sigma (\mathrm{Conv}2D(X_{H}^{\prime }))}\\ \;+\bar{X_{W}⊙\sigma (\mathrm{Conv}2D(X_{W}^{\prime }))})\\ \;=\frac{1}{3}(\bar{X_{C}⊙w_{C}}+\bar{X_{H}⊙w_{H}}+\bar{X_{W}⊙w_{W}}) \end{array}$$

where $w_{C}\in {\mathbb{R}}^{T\times 1\times H\times W}$, $w_{H}\in {\mathbb{R}}^{T\times 1\times C\times W}$, and $w_{W}\in {\mathbb{R}}^{T\times 1\times C\times H}$ denote the attention map generated by the left, middle, and right branches. σ denotes the sigmoid activation function. ⊙ is the element-wise multiplication. $\bar{X_{C}⊙w_{C}}$, $\bar{X_{H}⊙w_{H}}$, and $\bar{X_{W}⊙w_{W}}$ denote the execution of dimensional permuting operation on ***X***__*C*__⊙*w*__*C*__, ***X***__*H*__⊙*w*__*H*__, and ***X***__*W*__⊙*w*__*W*__ to retain the original input shape of *X*∈ℝ^*C*×*T*×*H*×*W*^. *Y*∈ℝ^*C*×*T*×*H*×*W*^ denotes the final output of the TSCI module.

## 3 Experiment

### 3.1 Datasets

In order to comprehensively evaluate the performance of various methods on basketball player action recognition, we adopted two popular basketball action recognition datasets to evaluate extensively. We will describe these two datasets in detail next.

**Figure 4:**
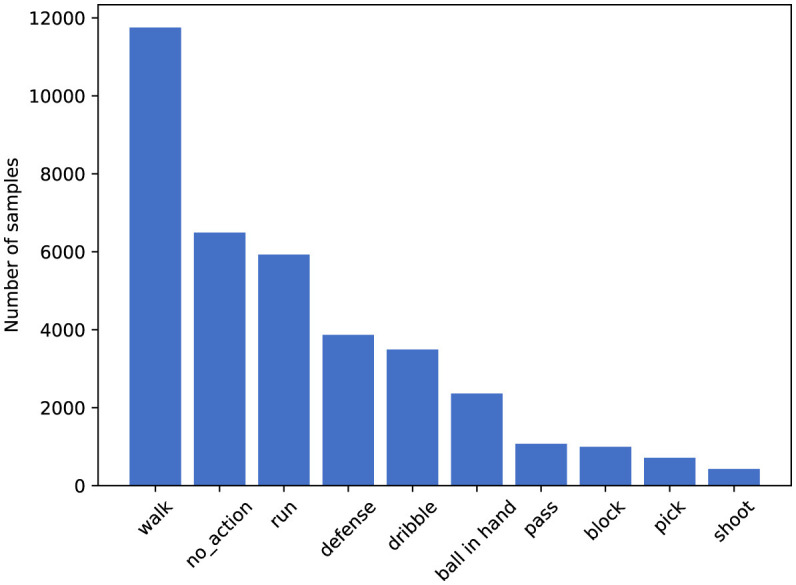
Number of samples per class on SpaceJam dataset.

**Figure 5:**
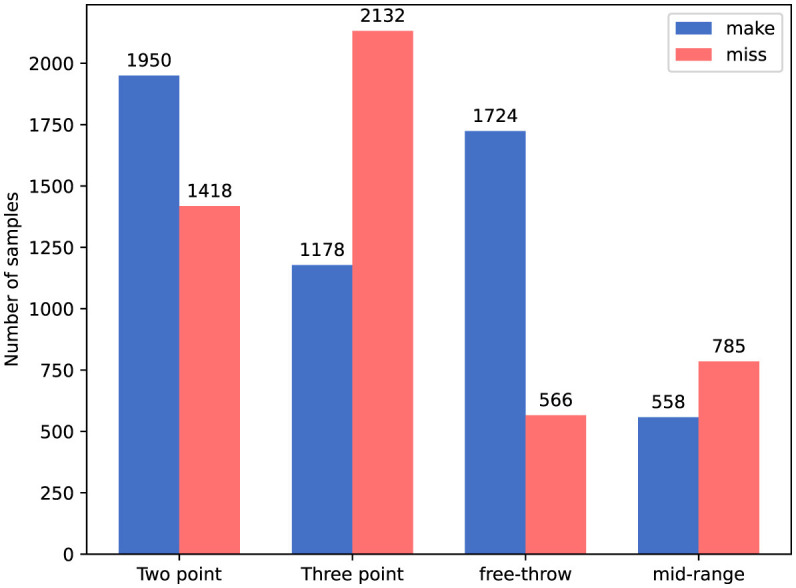
Number of samples per class on Basketball-51 dataset.

- **SpaceJam** (Francia et al., 2018). SpaceJam dataset is currently the most popular and accessible dataset available for the recognition of basketball players' movements. The dataset is divided into two parts: the first part consists of video clips in MP4 format, and the second part consists of the joint coordinates data of the athletes corresponding to each video. In this paper, we only utilize the video data from the first part. These videos originate from 15 segments of NBA championship and Italian championship basketball matches, with each video lasting 1.5 h. The dataset contains approximately 37,085 video clips in total, each comprising 16 frames, with each frame including three channels (RGB) and a resolution of 176 × 128 pixels. This dataset encompasses categories of 10 types of movements, namely: walk, no action, run, defense, dribble, ball in hand, pass, block, pick, and shoot. The distribution of data across different categories is illustrated in Figure 4.
- **Basketball-51** (Shakya et al., 2021). The videos in the Basketball-51 dataset originate from third-person perspective shots taken during media broadcasts of 51 NBA basketball games. The dataset comprises a total of 10,311 video clips, each standardized to 25 FPS, and each frame includes three channels (RGB) with a resolution of 320 × 240. The dataset contains 8 categories, which are: two-point miss (2p0), two-point make (2p1), three-point miss (3p0), three-point make (3p1), free throw miss (ft0), free throw make (ft1), mid-range shot miss (mp0), and mid-range shot make (mp1). The distribution of data across these categories is shown in Figure 5.

### 3.2 Evaluation metrics

To facilitate quantitative benchmarking of our and other methods, we select five standard metrics to assess the performance of various methods: accuracy, precision, recall, F1-Score, and AUC (area under the ROC curve). The formulas and variable explanations for each metric are as follows.

1. The formula of accuracy is shown in Equation 9:

(9) $$\begin{array}{l} Accuracy=\frac{TP+TN}{TP+TN+FP+FN} \end{array}$$

where TP indicates the number of true positives, TN represents the number of true negatives, FP is the number of false positives, and FN represents the number of false negatives.
2. The formula of precision is shown in Equation 10:

(10) $$\begin{array}{l} Precision=\frac{TP}{TP+FP} \end{array}$$

where TP indicates the number of true positives, and FP is the number of false positives.
3. The formula of recall is shown in Equation 11:

(11) $$\begin{array}{l} Recall=\frac{TP}{TP+FN} \end{array}$$

where TP indicates the number of true positives, and FN represents the number of false negatives.
4. The formula of F1-Score is shown in Equation 12:

(12) $$\begin{array}{l} F1\text{-}Score=2\times \frac{Precision\times Recall}{Precision+Recall} \end{array}$$

where Precision and Recall are defined as Equations 10, 11.
5. The formula of Area Under Curve (AUC) is shown in Equation 13:

(13) $$\begin{array}{l} AUC=\int_{0}^{1}ROC\left(x\right)dx \end{array}$$

where ROC(x) represents the relationship between the true positive rate and the false positive rate when x is the threshold.

### 3.3 Experimental setup

For the SpaceJam dataset, we directly use the original number of frames and frame size of each video clip in the dataset, so the shape of the input is *N*×3 × *T*×176 × 128, where *N* is the batch size, *T* denotes the number of frames of each video clip, *H* and *W* denotes the height and width of each frame. In this work, *N* is set to 16, and *T* is 8. For the Basketball-51 dataset, the cropped frames are resized to 120 × 160 for training the networks. The number of frames of each video clip *T* is set to 50. For all datasets, 80% of the dataset is divided into a training set, 10% into a validating set and 10% into a testing set. For fairness, we used the unified dataset splitting strategy for all methods. We train all the models on the training set, while observing the loss function curves on the validation set to avoid model overfitting, and finally test the models on the test set for various types of metrics, as reported in the results in Section 3.4.

As show in Table 1, we show the details of the state-of-the-art models to be compared with in this work. Each model was implemented using the author's open-source code and initialized using its pre-trained weights. Then we trained each model on the SpaceJam and Basketball-51 datasets. Specifically, ACA-Net is initialized with pre-trained weights on ImageNet to speed up the convergence of the network. For all experiments, we initially set the number of epochs to 30, with early stopping based on validation loss to prevent overfitting. The batch size is set to 16, and the learning rate is set at 0.0001. The total number of parameters in the model is approximately 26.8 million. We use the Adam optimizer (Kingma and Ba, 2014) for training on 8 NVIDIA GeForce RTX 3090 GPUs.

**Table 1:** The details of the state-of-the-art models.

**Model**	**Backbone**	**Pre-training**
C3D (Tran et al., 2015)	VGG-16	Sports-1M
R3D (Hara et al., 2018)	ResNet50	ImageNet
I3D (Carreira and Zisserman, 2017)	ResNet50	ImageNet
R(2+1)D (Tran et al., 2018)	ResNet50	Kinetics-400
SlowFast (Feichtenhofer et al., 2019)	ResNet50	Kinetics-400
TSN (Wang et al., 2016)	BN-Inception	ImageNet
TSM (Lin et al., 2019)	ResNet50	ImageNet
ViViT (Arnab et al., 2021)	ViT	ImageNet
Video-Swin (Liu et al., 2022)	Swin Transformer	ImageNet
**ACA-Net (Ours)**	ResNet50	ImageNet

### 3.4 Experimental results and analysis

#### 3.4.1 Classification results

In this section, we compared ACA-Net with the current mainstream action recognition methods on SpaceJam and Basketball-51 datasets to validate the effectiveness of the proposed method. As illustrated in Table 2, we present the experiment results conducted on SpaceJam dataset, comparing various state-of-the-art methods across key performance metrics. In this analysis, we evaluated accuracy, precision, recall, F1-Score, and AUC to comprehensively assess the efficiency and effectiveness of each method.

**Table 2:** Comparison with state-of-the-art methods on SpaceJam dataset.

**Methods**	**SpaceJam Dataset**				
	**Accuracy**	**Precision**	**Recall**	**F1-Score**	**AUC**
C3D (Tran et al., 2015)	64.11	65.81	46.34	50.75	90.80
R3D (Hara et al., 2018)	73.55	74.71	64.97	68.35	95.50
I3D (Carreira and Zisserman, 2017)	78.16	76.88	76.41	76.20	7.04
R(2+1)D (Tran et al., 2018)	75.60	77.17	71.78	73.70	96.34
SlowFast (Feichtenhofer et al., 2019)	63.98	59.81	55.95	57.44	92.04
TSN (Wang et al., 2016)	77.19	78.49	64.88	67.75	96.96
TSM (Lin et al., 2019)	81.21	83.90	77.08	79.68	97.98
ViViT (Arnab et al., 2021)	78.59	81.87	75.17	77.43	97.19
Video-Swin (Liu et al., 2022)	79.99	81.07	76.21	78.18	96.39
**ACA-Net (Ours)**	**89.26**	**90.85**	**88.91**	**89.78**	**98.99**

The bold values represent the optimal values for the respective metrics.

As it is clear, our proposed ACA-Net achieves more outstanding performance compared to other existing models for basketball action recognition in all evaluation criteria. ACA-Net achieves the accuracy rate of 89.26 for SpaceJam. The results indicate that the combination of LSTA module and TSCI module increased the recognition accuracy remarkably. 3D convolution-based approaches have limited receptive fields and they have simple structures relatively, which limit their capabilities for capturing the connections between distant frames and extracting discriminative features. Similarly, models like R(2+1)D and SlowFast, although effective to some extent, still fall short in leveraging temporal and spatial dynamics comprehensively, as evidenced by their lower F1-Scores and Recall values. The LSTA module in ACA-Net is capable of generating video-related adaptive convolutional kernels based on global temporal information, which can effectively capture temporal contextual features in basketball videos. Moreover, the TSCI module in ACA-Net can conduct cross-dimensional information interaction in the spatial and channel dimensions to extract discriminative features more effectively.

**Figure 6:**
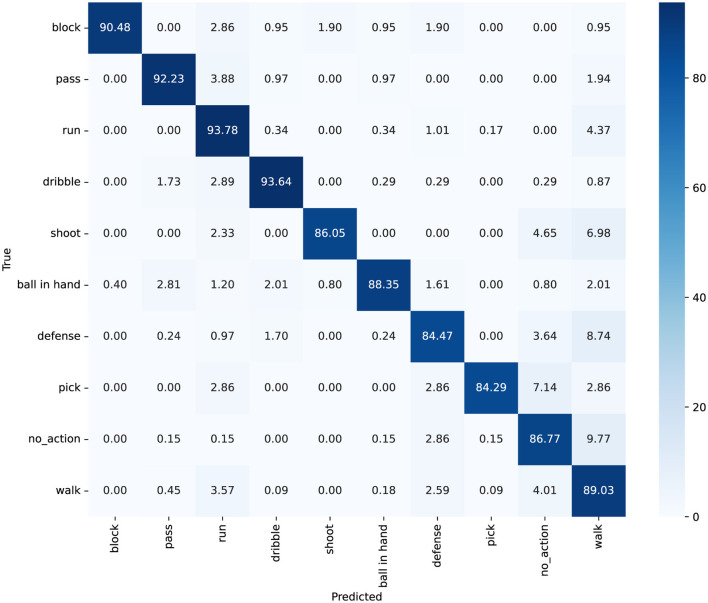
The confusion matrix for classification on the SpaceJam dataset, with values represented as percentages.

**Figure 7:**
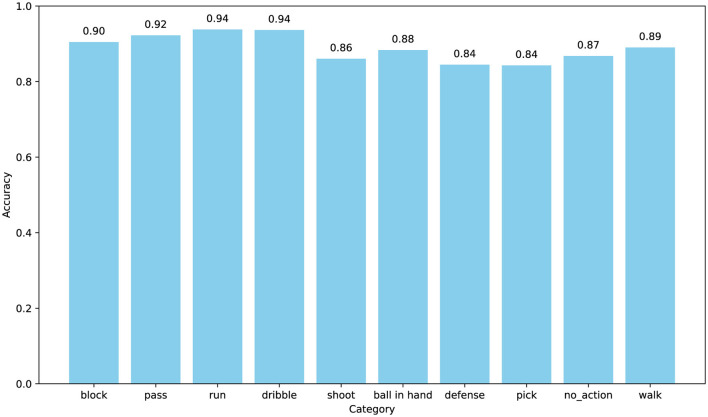
The accuracy for each category on the SpaceJam dataset.

Furthermore, we explored the model's accuracy for each action. The SpaceJam dataset encompasses categories of 10 types of actions, namely: walk, no action, run, defense, dribble, ball in hand, pass, block, pick, and shoot. The confusion matrix and accuracy of individual category for SpaceJam are shown in Figures 6, 7. Analyzing the experimental results, we observe several key performance metrics of ACA-Net. For the majority of actions, such as “block,” “pass,” and “run,” ACA-Net indicates a high recognition accuracy, evidenced by the diagonal dominance in these categories. Specifically, the model achieves recognition rates of 90% for “block” and “pass,” and 94% for “run,” indicating the model's robustness in identifying these actions. This performance underscores the efficacy of the LSTA module in capturing temporal dynamics, which are crucial for distinguishing between rapid, sequential actions in basketball.

Notably, ACA-Net also indicates exceptional performance in distinguishing between actions with subtle differences. The model's ability to classify “dribble” and “defense” actions, with high accuracy rates of 88% and 84% respectively, highlights the effectiveness of the TSCI module. This module's capability to enhance feature representation by learning the interactions between spatial and channel dimensions proves crucial in identifying nuanced variations in player movements and postures. Moreover, the model's precision in recognizing the “no_action” category, with 577 correct identifications, further validates the TSCI module's strength. By effectively capturing the distinguishing features of inactive moments amidst dynamic play, the TSCI module ensures that ACA-Net can accurately segregate active and inactive states, which is critical in a complex sports environment.

As illustrated in Table 3, we report the experiment results conducted on Basketball-51 dataset, comparing various state-of-the-art methods across key performance metrics, including Accuracy, Precision, Recall, F1-Score, and AUC. ACA-Net achieves an accuracy of 92.05%, substantially higher than the next best-performing model, Video-Swin, which has an accuracy of 85.08%. Additionally, the precision and recall rates of ACA-Net, at 90.44% and 89.39% respectively, indicate its remarkable ability to minimize false positives and false negatives, ensuring reliable action recognition. The F1-Score of 89.68% further corroborates the balanced performance of ACA-Net across all evaluation metrics. Moreover, the AUC metric, standing at 99.17%, indicates ACA-Net's good overall performance in distinguishing between different categories. This indicates that the TSCI module's ability to extract distinguishing features is particularly effective, ensuring high separability between various basketball actions.

**Table 3:** Comparison with state-of-the-art methods on Basketball-51 dataset.

**Methods**	**Basketball-51 Dataset**				
	**Accuracy**	**Precision**	**Recall**	**F1-Score**	**AUC**
C3D (Tran et al., 2015)	62.60	57.29	52.88	53.21	87.65
R3D (Hara et al., 2018)	77.91	73.64	67.07	68.12	96.69
I3D (Carreira and Zisserman, 2017)	75.78	75.85	63.60	64.83	96.22
R(2+1)D (Tran et al., 2018)	72.67	70.38	62.24	62.56	94.70
SlowFast (Feichtenhofer et al., 2019)	59.50	54.15	49.82	50.40	89.13
TSN (Wang et al., 2016)	66.09	53.97	51.20	47.80	92.69
TSM (Lin et al., 2019)	79.07	76.12	73.74	74.64	95.72
ViViT (Arnab et al., 2021)	84.88	67.13	65.77	66.31	97.90
Video-Swin (Liu et al., 2022)	85.08	85.23	80.51	81.22	97.64
**ACA-Net (Ours)**	**92.05**	**90.44**	**89.39**	**89.68**	**99.17**

The bold values represent the optimal values for the respective metrics.

**Figure 8:**
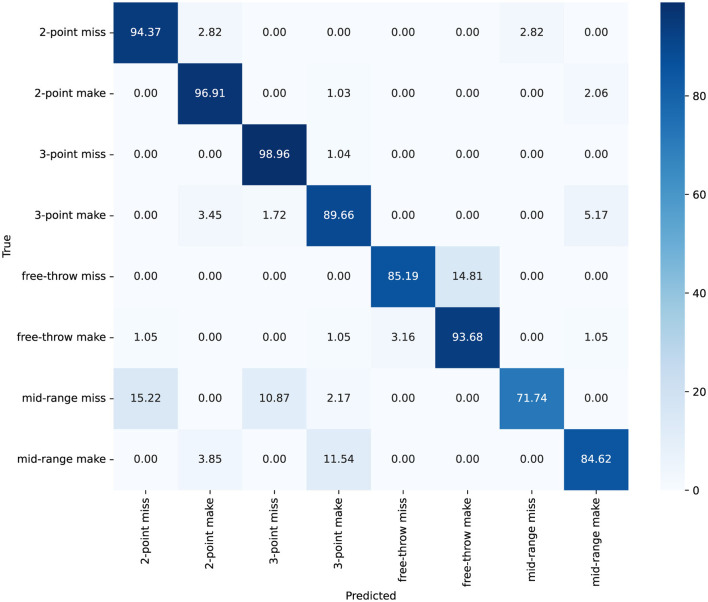
The confusion matrix for classification on the Basketball-51 dataset, with values represented as percentages.

**Figure 9:**
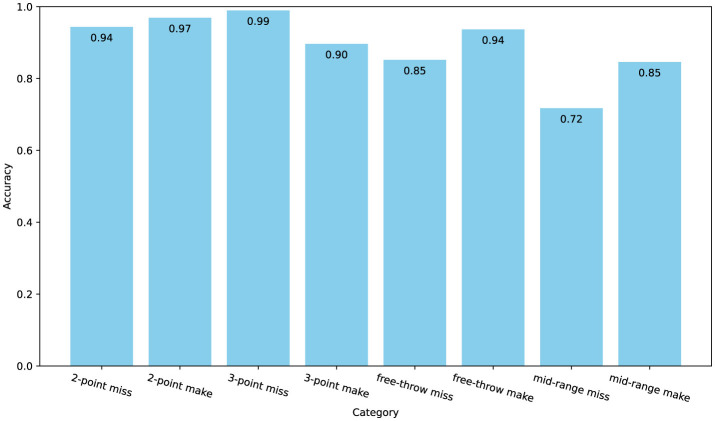
The accuracy for each category on the Basketball-51 dataset.

As shown in Figures 8, 9, the confusion matrix indicates high classification accuracy for most categories. For instance, “2-point make” and “3-point miss” actions are classified with accuracies of 97% and 99% correct predictions respectively, demonstrating ACA-Net's robustness in identifying these frequent basketball actions. Furthermore, the ACA-Net indicates good performance in recognizing successful actions, such as “3-point make” and “free-throw make,” with only minor misclassifications. Specifically, “3-point make” has 52 correct identifications with just a few misclassifications. Additionally, the model maintains substantial accuracy in less frequent and more challenging actions such as “mid-range make” and “free-throw miss,” with accuracies of 85%. This indicates ACA-Net's versatility and robustness in handling a diverse set of actions, further validated by its overall high performance across different metrics.

In summary, ACA-Net's advanced design, incorporating the LSTA and TSCI modules, equips it with a robust feature extraction capability that significantly outperforms existing state-of-the-art methods on the SpaceJam and Basketball-51 datasets. This underscores ACA-Net's potential as a powerful tool for accurate and reliable basketball action recognition in complex scenarios.

#### 3.4.2 Feature visualization

**Figure 10:**
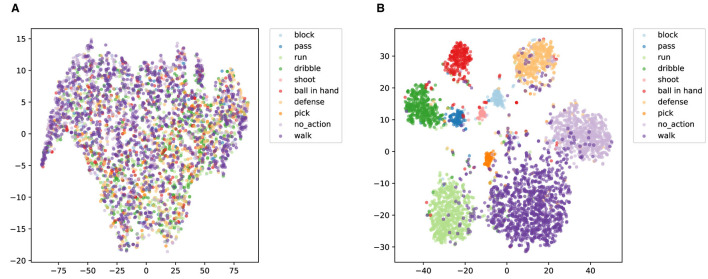
t-SNE visualization of ACA-Net for SpaceJam test set. **(A)** Before training. **(B)** After training.

t-SNE (Belkina et al., 2019) is a probabilistic-based nonlinear dimensionality reduction technique that learns a mapping by minimizing the Kullback-Leibler scatter between data points in the high and low dimensional spaces. t-SNE is particularly suited to the visualization of data as it reveals the underlying clustering structure in a dataset. In this work, we explore the differences in feature distribution of ACA-Net before and after training for the SpaceJam and Basketball-51 datasets. Figure 10 illustrates the t-SNE visualization of ACA-Net for SpaceJam test set. The t-SNE plot before training (Figure 10A) indicates a scattered and poorly separated feature space. Most action categories overlap significantly, with no clear boundaries between different classes. This overlap suggests that the model's initial feature extraction capability is inadequate, failing to distinguish between various basketball actions.

In contrast, the t-SNE visualization after training (Figure 10B) indicates a well-organized feature space with distinct clusters for each action category. The LSTA module's ability to capture global and local temporal features, combined with the TSCI module's enhancement of spatial-channel interactions, has led to a more discriminative feature space. The reduced overlap and increased separation between clusters indicate that the trained ACA-Net model can effectively differentiate between various basketball actions. Despite the overall improvement in feature discrimination after training, the t-SNE visualization (Figure 10B) reveals some residual misclassification issues. Several samples from other action categories are misclassified or overlap with the “walk” cluster. The reason for this problem may be that the distribution of action categories in the SpaceJam dataset is unbalanced, with “walking” potentially being a more common action. This imbalance could bias the model toward overfitting on more frequent actions, leading to higher misclassification rates for less frequent or more complex actions.

**Figure 11:**
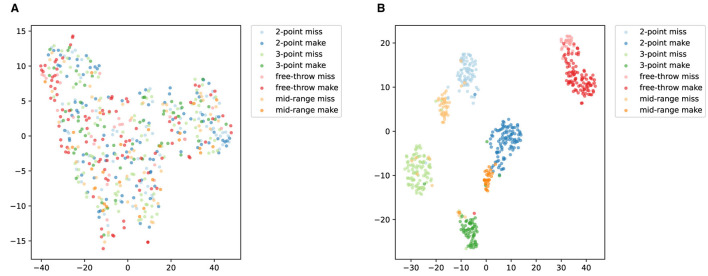
t-SNE visualization of ACA-Net for Basketball-51 test set. **(A)** Before training. **(B)** After training.

Figure 11 illustrates the t-SNE visualization of ACA-Net for Basketball-51 test set. The t-SNE plot before training (Figure 11A) displays a scattered and poorly differentiated feature space. The majority of action categories overlap considerably, with minimal distinct clustering. This suggests that the initial features extracted by the model lack the discriminative power necessary to effectively differentiate between the various types of basketball actions. The overlap indicates an inadequacy in capturing the unique characteristics of each action category.

The t-SNE visualization after training (Figure 11B) shows a markedly improved organization of the feature space, with more distinct clusters for each action category. The ACA-Net's LSTA and TSCI modules have evidently enhanced the feature representations. The LSTA module's ability to learn both global and local temporal features, coupled with the TSCI module's enhancement of spatial-channel interactions, has resulted in a feature space where action categories are more clearly separated. This improved separation indicates a successful learning of the distinguishing features for each basketball action type. Despite the overall improvement, certain action categories, such as “2-point miss” and “mid-range miss,” exhibit some degree of overlap or proximity. The reason for this may be that the imbalance in the samples of the dataset introduces a bias in the performance of the model. Additionally, the actions “2-point miss” and “mid-range miss” may share similar visual and temporal characteristics, especially in the dynamics of the shot motion and the outcome of the action. The difficulty in differentiating these subtle differences can lead to misclassification.

#### 3.4.3 Ablation experiment

The ablation studies are performed on SpaceJam and Basketball-51 datasets to investigate the effects of LSTA and TSCI modules on the model performance. The baseline model is the ResNet50 (He et al., 2016) with original architecture and without LSTA and TSCI modules. We explored the effect of adding only one module and adding both modules on model performance separately. As shown in Table 4, the ablation study results indicate the significant impact of both the LSTA and TSCI modules on the performance of the ACA-Net model. The addition of the LSTA module to the baseline model results in substantial performance improvements, increasing accuracy to 86.87% and the F1-score to 87.93% on the SpaceJam dataset, and achieving 88.17% accuracy and 84.93% F1-score on the Basketball-51 dataset. Similarly, the integration of the TSCI module enhances the baseline model's accuracy to 85.06% and F1-score to 85.31% on SpaceJam, and to 84.30% accuracy and 80.93% F1-score on Basketball-51. The combined use of both modules yields the highest performance, with the model achieving 89.26% accuracy and 89.78% F1-score on SpaceJam, and 92.05% accuracy and 89.68% F1-score on Basketball-51. These results indicate that the LSTA and TSCI modules complement each other, effectively leveraging temporal, spatial, and channel-level features to improve action recognition performance.

**Table 4:** Ablation experiments of LSTA and TSCI modules.

**Model**	**Dataset**			
	**SpaceJam**		**Basketball-51**	
	**Accuracy**	**F1-Score**	**Accuracy**	**F1-Score**
Baseline	80.97	82.34	83.72	77.67
Baseline + LSTA	86.87	87.93	88.17	84.93
Baseline + TSCI	85.06	85.31	84.30	80.93
Baseline + LSTA + TSCI	**89.26**	**89.78**	**92.05**	**89.68**

The bold values represent the optimal values for the respective metrics.

## 4 Discussion

The results presented in this study indicate the superior performance of the proposed ACA-Net for basketball action recognition on the SpaceJam and Basketball-51 datasets. The integration of the LSTA module and the TSCI module has proven effective in extracting comprehensive features across temporal, spatial, and channel dimensions. The experimental results reveal that ACA-Net achieves higher accuracy, precision, recall, F1-Score, and AUC compared to existing state-of-the-art methods. This marked improvement can be attributed to the combined strengths of the LSTA and TSCI modules. The LSTA module enhances the model's capability to capture both global and local temporal features, which is particularly important for recognizing rapid and sequential actions that are common in basketball. This dual focus on temporal scales allows ACA-Net to maintain robustness even in the presence of actions that are subtle or occur in quick succession. The TSCI module further contributes by learning interaction features between spatial and channel dimensions, significantly boosting the feature representation's discriminative power. This cross-dimensional interaction is critical for differentiating between visually similar actions, a common challenge in sports video analysis.

Despite ACA-Net indicates promising results, several research avenues remain open for further exploration. First, exploring the scalability of ACA-Net to larger and more diverse datasets. This would provide valuable insights into its generalizability and robustness across different settings and conditions, such as varying game strategies, player behaviors, and environmental factors. Additionally, integrating more contextual information, such as player positions, game scenarios, and team strategies, could further enhance the model's performance by providing richer, more informative features for action recognition.

Another critical area for future work involves the real-time implementation and optimization of ACA-Net for deployment in live sports analytics systems. Such advancements would allow for immediate action recognition and analysis, providing valuable insights to coaches, players, and referees during games. Exploring ACA-Net's application beyond basketball to other sports and activity recognition tasks would also validate its versatility and adaptability across different domains. Finally, collaborative research efforts that incorporate multi-modal data integration, combining visual data with inputs from sensors, audio, or even physiological signals, could open new frontiers in the development of comprehensive and robust action recognition systems.

## 5 Conclusions

Neural network models have become a cornerstone in the development of autonomous robotic systems, enabling machines to perceive, analyze, and respond to complex environments with increasing sophistication. These models, inspired by the structure and function of the human brain, are driving innovations in robotics by enhancing adaptability, decision-making, and multi-modal data processing. In this paper, we proposed an adaptive context-aware network, ACA-Net, designed specifically for basketball action recognition, as an example of how such neural network architectures can address real-world challenges in dynamic environments. The network's architecture integrates LSTA and TSCI modules, each targeting different aspects of video feature extraction to enhance overall performance. The LSTA module enhances feature representation through a gated convolutional layer, followed by the complementary learning of temporal context features via the local branch's importance weights and the global branch's adaptive convolutions. In parallel, the TSCI module improves the extraction of discriminative features by facilitating cross-dimensional interactions between spatial and channel features through its three-branch structure. The fusion of long short-term temporal information from the LSTA module with spatial-channel interaction information from the TSCI module, guided by global contextual information, enables ACA-Net to effectively discriminate between different basketball actions.

The effectiveness of ACA-Net was validated through extensive comparisons with current state-of-the-art methods, demonstrating its superior performance on the SpaceJam and Basketball-51 datasets, achieving accuracy rates of 89.26% and 92.05%, respectively. Additionally, dimensionality reduction and visualization of the features extracted by ACA-Net, performed using t-SNE, highlighted the model's strong discriminative capability across various action categories. Ablation experiments further validated the effectiveness of each module within ACA-Net, underscoring their contributions to the overall performance of the network.

These findings indicate that ACA-Net has made notable progress in the field of sports action recognition. By addressing the specific challenges of basketball action recognition, such as complex backgrounds, subtle differences in actions, and inconsistent lighting. Looking ahead, ACA-Net holds potential for broader applications in autonomous robotics, where precise action recognition is crucial for decision-making in dynamic and unstructured environments. Future research should focus on optimizing ACA-Net for real-time deployment, exploring its integration with multi-modal sensory data, and assessing its applicability in various autonomous robotic tasks. By extending ACA-Net's capabilities, we can further contribute to the advancement of neural network models in the robotics domain, enhancing both robotic autonomy and human-robot collaboration.

## Data Availability

The raw data supporting the conclusions of this article will be made available by the authors, without undue reservation.
