# Supplementary material for: ACA-Net: adaptive context-aware network for basketball action recognition
Source: Front Neurorobot. 2024 Sep 25;18:1471327. doi: 10.3389/fnbot.2024.1471327 (PMC11461453; doi:10.3389/fnbot.2024.1471327)
Supplement: Supplementary file 1 [file Data_Sheet_1.ZIP › PDF examples/Frontiers.pdf]

Article Title

First Author [1,*], **Co-Author** [2] **and Co-Author** [1,2]

[1] *Laboratory X, Institute X, Department X, Organization X, City X , State XX (only USA, Canada and Australia), Country X*

[2] *Laboratory X, Institute X, Department X, Organization X, City X , State XX (only USA, Canada and Australia), Country X*

Correspondence*:
Corresponding Author
email@uni.edu

ABSTRACT

For full guidelines regarding your manuscript please refer to Author Guidelines.

As a primary goal, the abstract should render the general significance and conceptual advance of the work clearly accessible to a broad readership. References should not be cited in the abstract. Leave the Abstract empty if your article does not require one, please see Summary Table for details according to article type.

Keywords: keyword, keyword, keyword, keyword, keyword, keyword, keyword, keyword

1 INTRODUCTION

For Original Research Articles (Name et al., 1996), Clinical Trial Articles (LastName1 et al., 2013), and Technology Reports (Surname1, 2010), the introduction should be succinct, with no subheadings (Name, 1993). For Case Reports the Introduction should include symptoms at presentation (Surname, 2002), physical exams and lab results (LastName1 et al., 2011).

2 ARTICLE TYPES

For requirements for a specific article type please refer to the Article Types on any Frontiers journal page. Please also refer to Author Guidelines for further information on how to organize your manuscript in the required sections or their equivalents for your field

3 MANUSCRIPT FORMATTING

3.1 Heading Levels

3.2 Level 2

3.2.1 Level 3

3.2.1.1 Level 4

3.2.1.1.1 Level 5

3.3 Equations

Equations should be inserted in editable format from the equation editor.

$$\sum x + y = Z \tag{1}$$

3.4 Figures

Frontiers requires figures to be submitted individually, in the same order as they are referred to in the manuscript. Figures will then be automatically embedded at the bottom of the submitted manuscript. Kindly ensure that each table and figure is mentioned in the text and in numerical order. Figures must be of sufficient resolution for publication see here for examples and minimum requirements. Figures which are not according to the guidelines will cause substantial delay during the production process. Please see here for full figure guidelines. Cite figures with subfigures as figure 2a and 2b.

3.4.1 Permission to Reuse and Copyright

Figures, tables, and images will be published under a Creative Commons CC-BY licence and permission must be obtained for use of copyrighted material from other sources (including re-published/adapted/modified/partial figures and images from the internet). It is the responsibility of the authors to acquire the licenses, to follow any citation instructions requested by third-party rights holders, and cover any supplementary charges.

3.5 Tables

Tables should be inserted at the end of the manuscript. Please build your table directly in LaTeX.Tables provided as jpeg/tiff files will not be accepted. Please note that very large tables (covering several pages) cannot be included in the final PDF for reasons of space. These tables will be published as Supplementary Material on the online article page at the time of acceptance. The author will be notified during the typesetting of the final article if this is the case.

3.6 International Phonetic Alphabet

To include international phonetic alphabet (IPA) symbols, please include the following functions: Under useful packages, include:

```
\usepackage{tipa}
```

In the main text, when inputting symbols, use the following format:

`\text[symbolname]`

e.g.

`\textgamma`

4 NOMENCLATURE

4.1 Resource Identification Initiative

To take part in the Resource Identification Initiative, please use the corresponding catalog number and RRID in your current manuscript. For more information about the project and for steps on how to search for an RRID, please click here.

4.2 Life Science Identifiers

Life Science Identifiers (LSIDs) for ZOOBANK registered names or nomenclatural acts should be listed in the manuscript before the keywords. For more information on LSIDs please see Inclusion of Zoological Nomenclature section of the guidelines.

5 ADDITIONAL REQUIREMENTS

For additional requirements for specific article types and further information please refer to Author Guidelines.

CONFLICT OF INTEREST STATEMENT

The authors declare that the research was conducted in the absence of any commercial or financial relationships that could be construed as a potential conflict of interest.

AUTHOR CONTRIBUTIONS

The Author Contributions section is mandatory for all articles, including articles by sole authors. If an appropriate statement is not provided on submission, a standard one will be inserted during the production process. The Author Contributions statement must describe the contributions of individual authors referred to by their initials and, in doing so, all authors agree to be accountable for the content of the work. Please see here for full authorship criteria.

FUNDING

Details of all funding sources should be provided, including grant numbers if applicable. Please ensure to add all necessary funding information, as after publication this is no longer possible.

ACKNOWLEDGMENTS

This is a short text to acknowledge the contributions of specific colleagues, institutions, or agencies that aided the efforts of the authors.

SUPPLEMENTAL DATA

Supplementary Material should be uploaded separately on submission, if there are Supplementary Figures, please include the caption in the same file as the figure. LaTeX Supplementary Material templates can be found in the Frontiers LaTeX folder.

DATA AVAILABILITY STATEMENT

The datasets [GENERATED/ANALYZED] for this study can be found in the [NAME OF REPOSITORY] [LINK].

REFERENCES

[Dataset] LastName1, A., LastName2, A., and LastName3, A. (2011). Data title. doi:10.000/55555

LastName1, A., LastName2, A., and LastName3, A. (2013). Article title. *Frontiers in Neuroscience* 30, 10127–10134. doi:10.3389/fnins.2013.12345

Name, A. (1993). *The title of the work* (The city: The name of the publisher)

Name, C., Surname, D., and LastName, F. (1996). The title of the work. In *The title of the conference proceedings*, eds. E. Name1 and E. Name2 (The name of the publisher), 41–50

Surname, B. (2002). The title of the work. In *The title of the book*, ed. E. Name (The city: The name of the publisher). 201–213

Surname1, H. (2010). *The title of the work* (Patent country: Patent number)

FIGURE CAPTIONS

Figure 1. Enter the caption for your figure here. Repeat as necessary for each of your figures

Figure 2a. This is Subfigure 1.

Figure 2b. This is Subfigure 2.

Figure 2. Enter the caption for your subfigure here. **(A)** This is the caption for Subfigure 1. **(B)** This is the caption for Subfigure 2.
