# Supplementary material for: ACA-Net: adaptive context-aware network for basketball action recognition
Source: Front Neurorobot. 2024 Sep 25;18:1471327. doi: 10.3389/fnbot.2024.1471327 (PMC11461453; doi:10.3389/fnbot.2024.1471327)
Supplement: Supplementary file 1 [file Data_Sheet_1.ZIP › PDF examples/Frontiers_SupplementaryMaterial.pdf]

Supplementary Material

1 SUPPLEMENTARY DATA

Supplementary Material should be uploaded separately on submission. Please include any supplementary data, figures and/or tables. All supplementary files are deposited to FigShare for permanent storage and receive a DOI.

Supplementary material is not typeset so please ensure that all information is clearly presented, the appropriate caption is included in the file and not in the manuscript, and that the style conforms to the rest of the article. To avoid discrepancies between the published article and the supplementary material, please do not add the title, author list, affiliations or correspondence in the supplementary files.

2 SUPPLEMENTARY TABLES AND FIGURES

For more information on Supplementary Material and for details on the different file types accepted, please see the Supplementary Material section of the Author Guidelines.

Figures, tables, and images will be published under a Creative Commons CC-BY licence and permission must be obtained for use of copyrighted material from other sources (including re-published/adapted/modified/partial figures and images from the internet). It is the responsibility of the authors to acquire the licenses, to follow any citation instructions requested by third-party rights holders, and cover any supplementary charges.

2.1 Figures

Figure S1. Enter the caption for your figure here. Repeat as necessary for each of your figures

A

B

Figure S2. This is a figure with sub figures, **(A)** is one logo, **(B)** is a different logo.
