# Supplementary figures and images for: ACA-Net: adaptive context-aware network for basketball action recognition
Source: Front Neurorobot. 2024 Sep 25;18:1471327. doi: 10.3389/fnbot.2024.1471327 (PMC11461453; doi:10.3389/fnbot.2024.1471327)

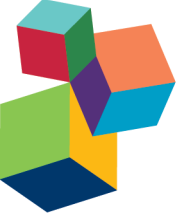

frontiers

Supplement: Supplementary file 1 [file Data_Sheet_1.ZIP › logo1.pdf]

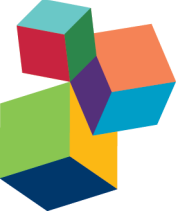

Supplement: Supplementary file 1 [file Data_Sheet_1.ZIP › logo2.pdf]

A

frontiers  
FOR YOUNG MINDS

B

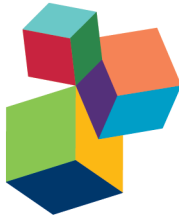

Supplement: Supplementary file 1 [file Data_Sheet_1.ZIP › logos.pdf]

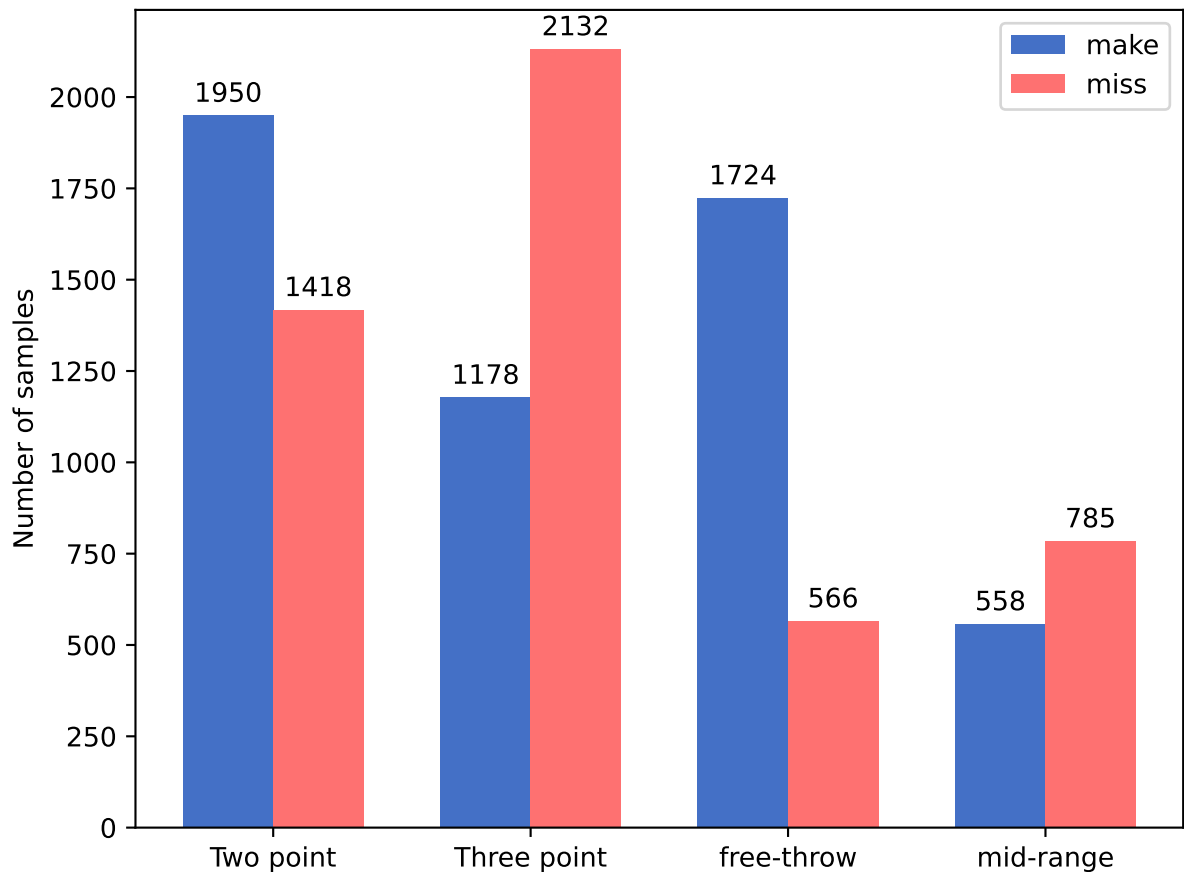

Supplement: Supplementary file 1 [file Data_Sheet_1.ZIP › figures/basketball-51.pdf]

Number of samples

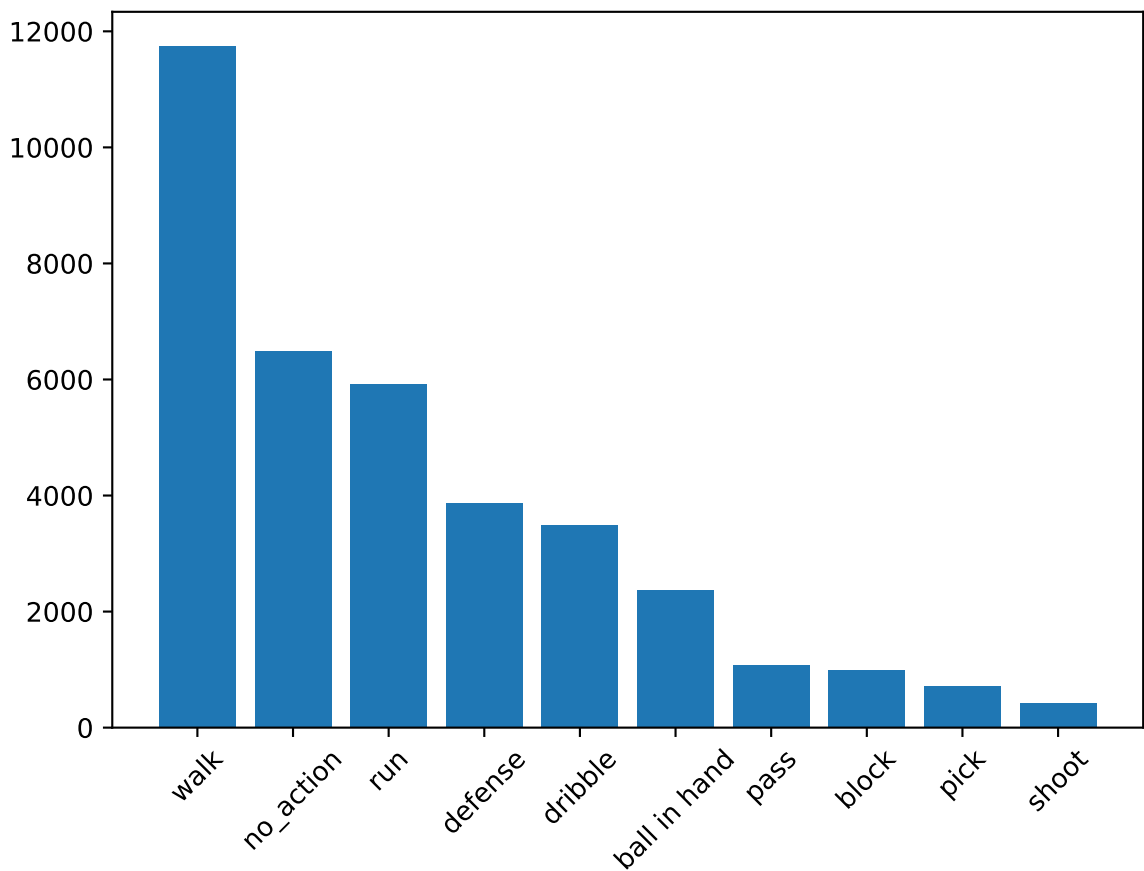

Supplement: Supplementary file 1 [file Data_Sheet_1.ZIP › figures/data_distribution.pdf]

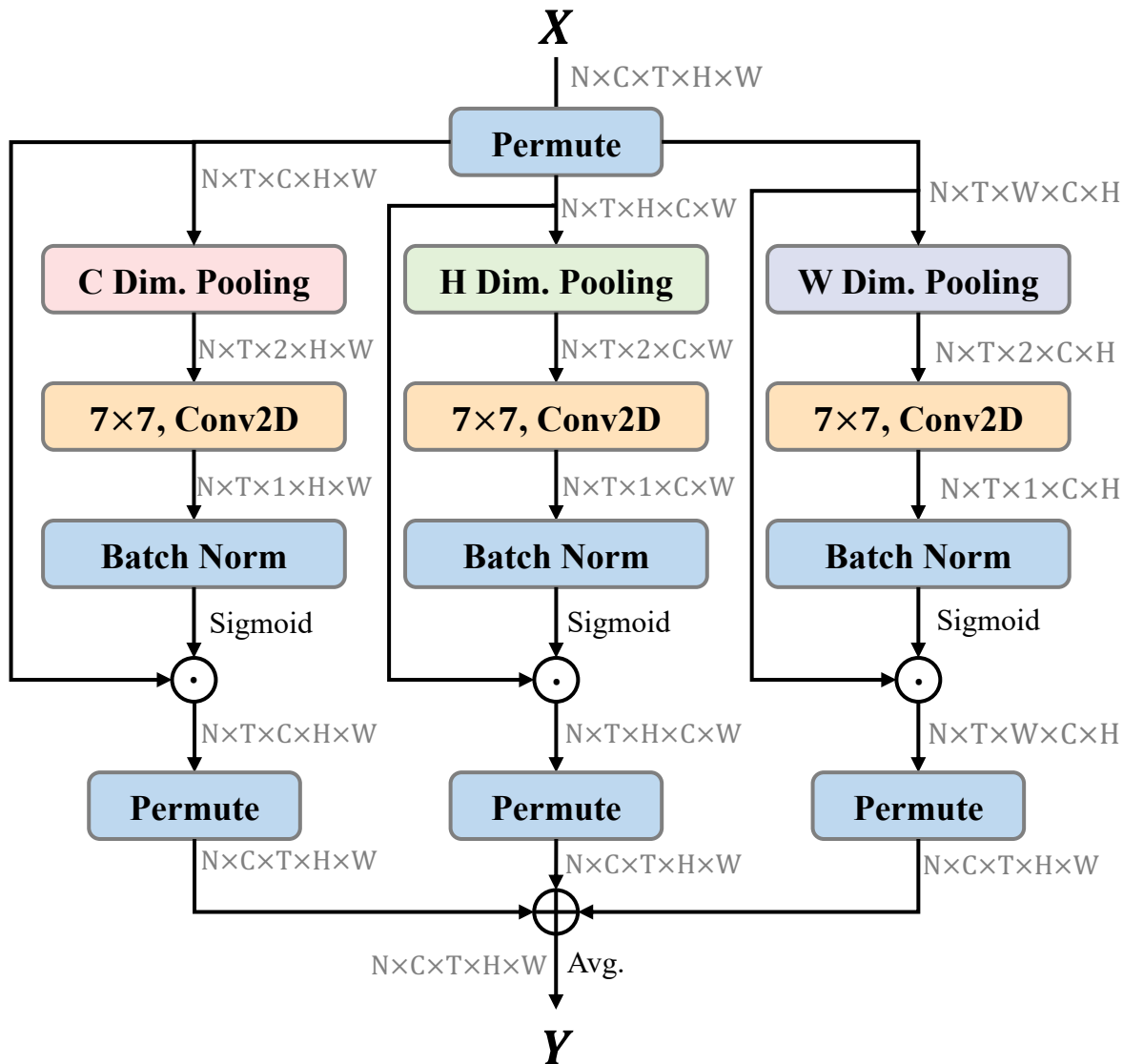

Supplement: Supplementary file 1 [file Data_Sheet_1.ZIP › figures/TSCI.pdf]

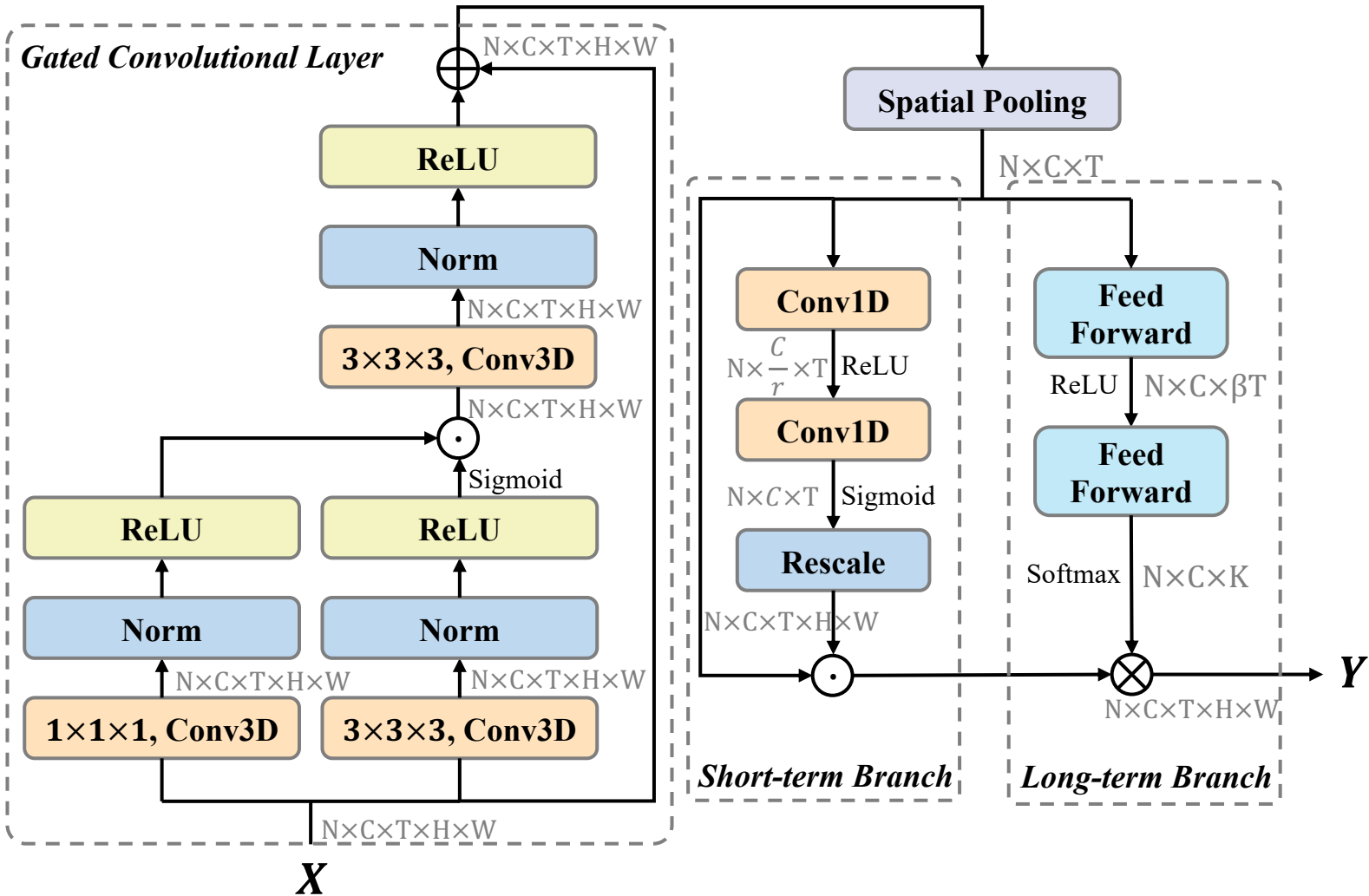

Supplement: Supplementary file 1 [file Data_Sheet_1.ZIP › figures/LSTA.pdf]

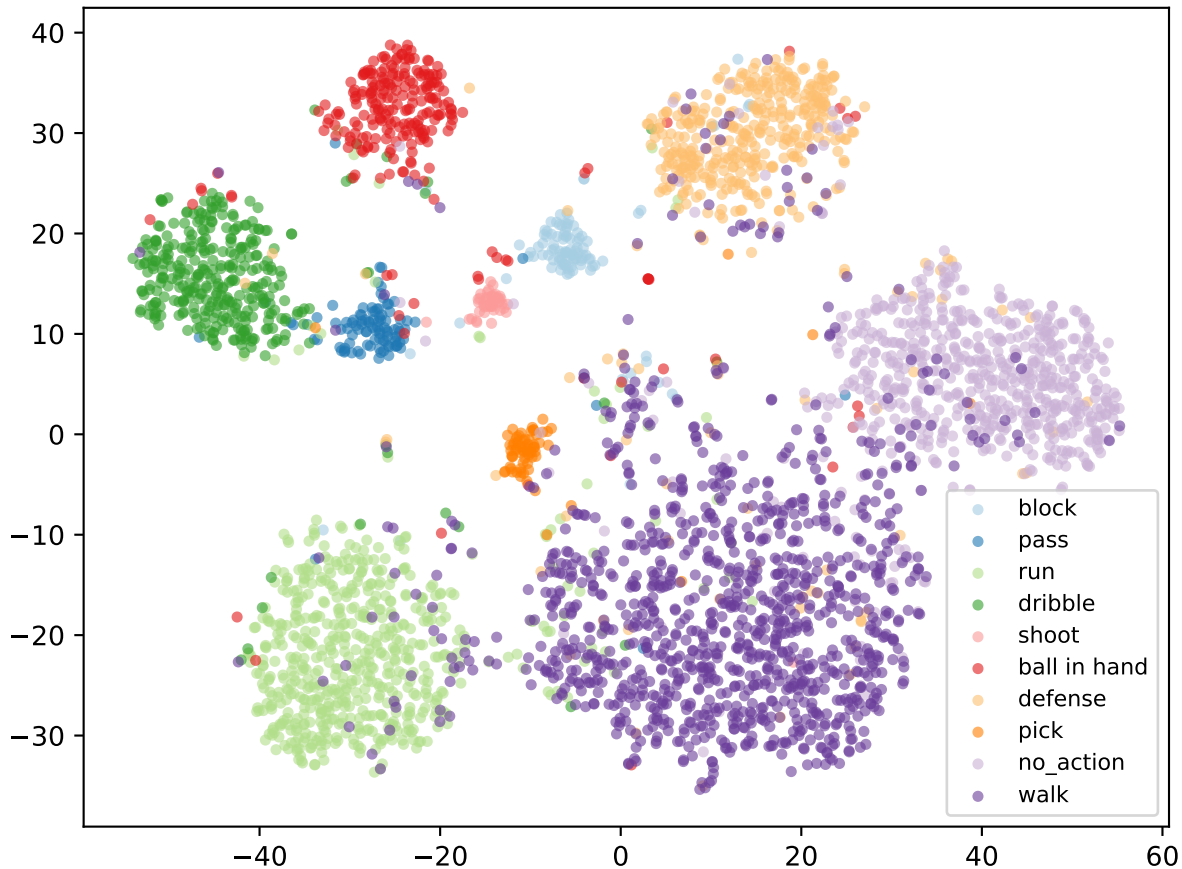

Supplement: Supplementary file 1 [file Data_Sheet_1.ZIP › figures/t_SNE_SpaceJam_30epoch.pdf]

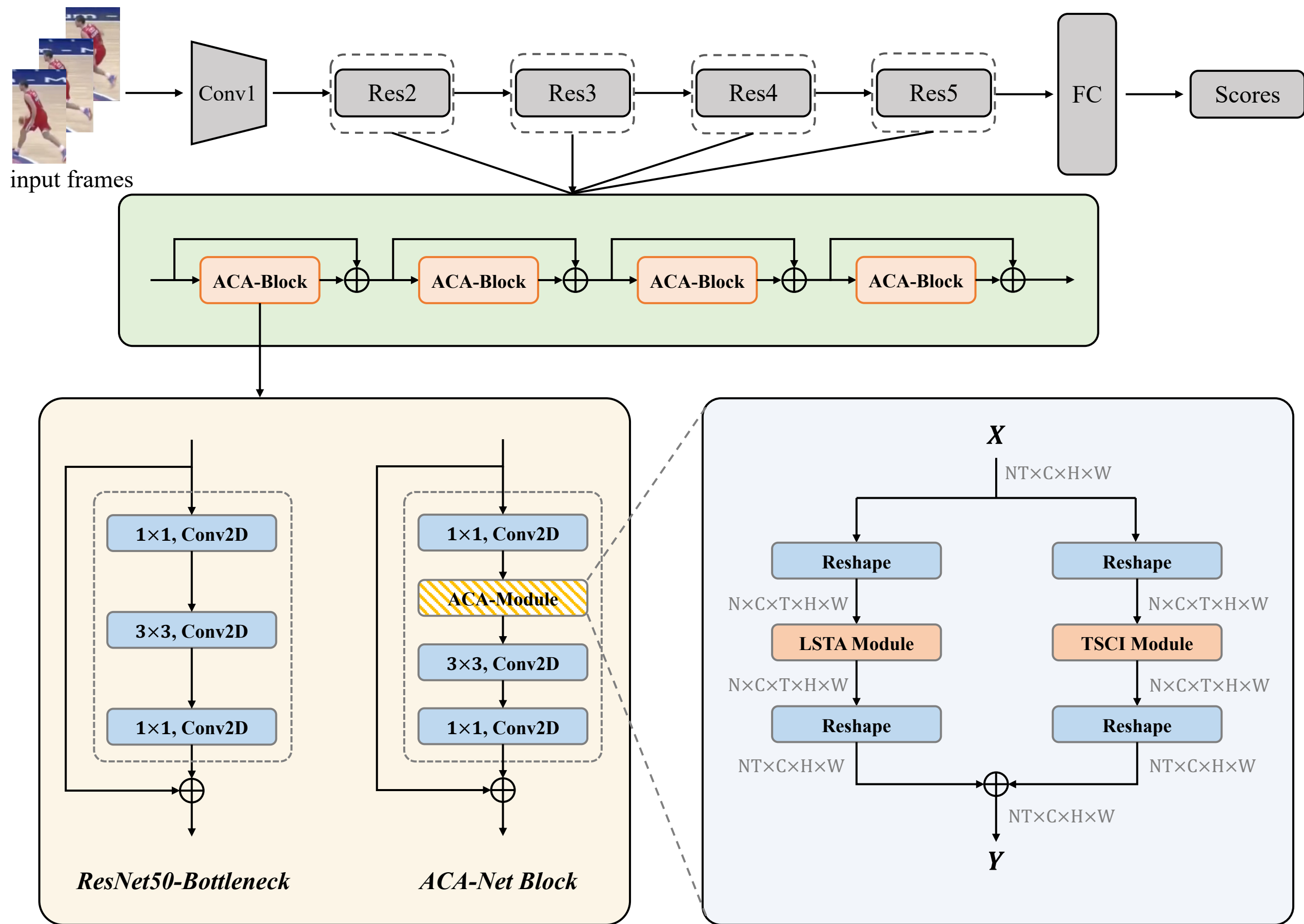

Supplement: Supplementary file 1 [file Data_Sheet_1.ZIP › figures/overview.pdf]

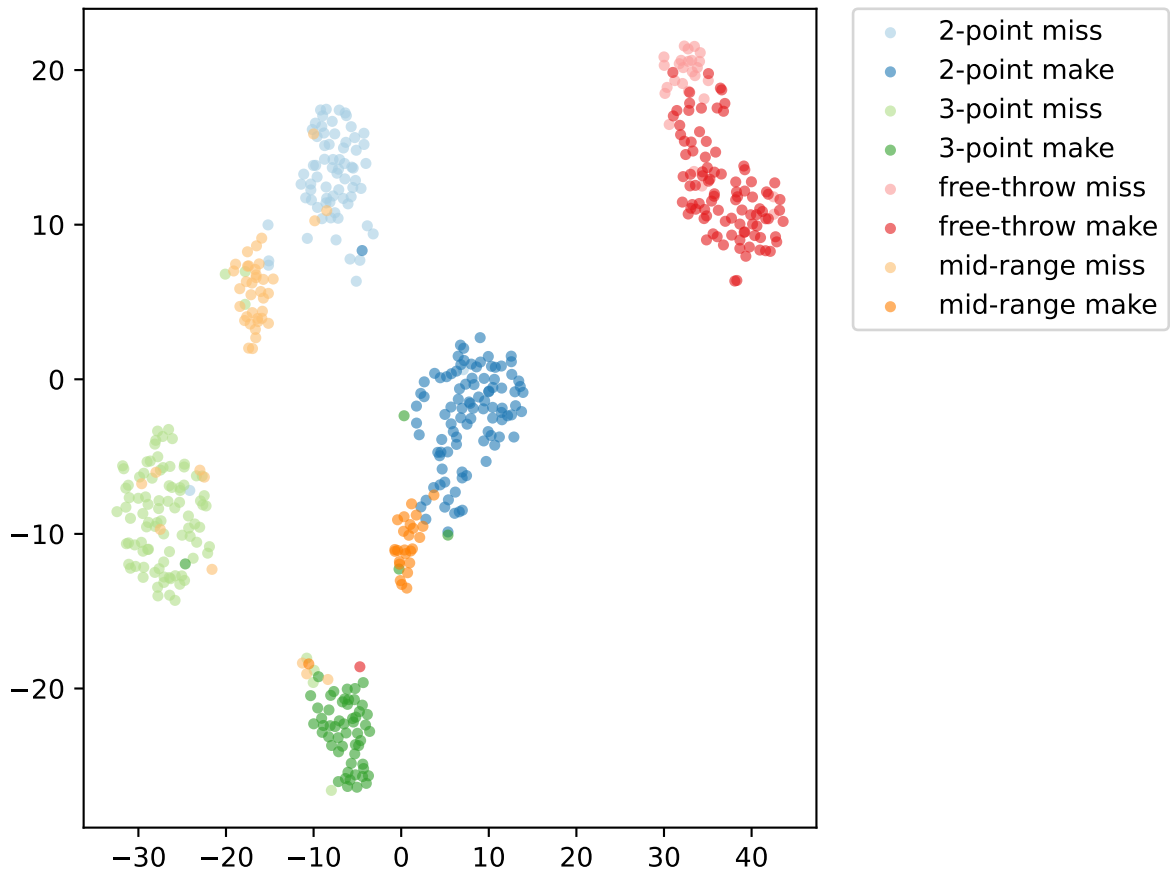

Supplement: Supplementary file 1 [file Data_Sheet_1.ZIP › figures/tsne_B51_after_training.pdf]

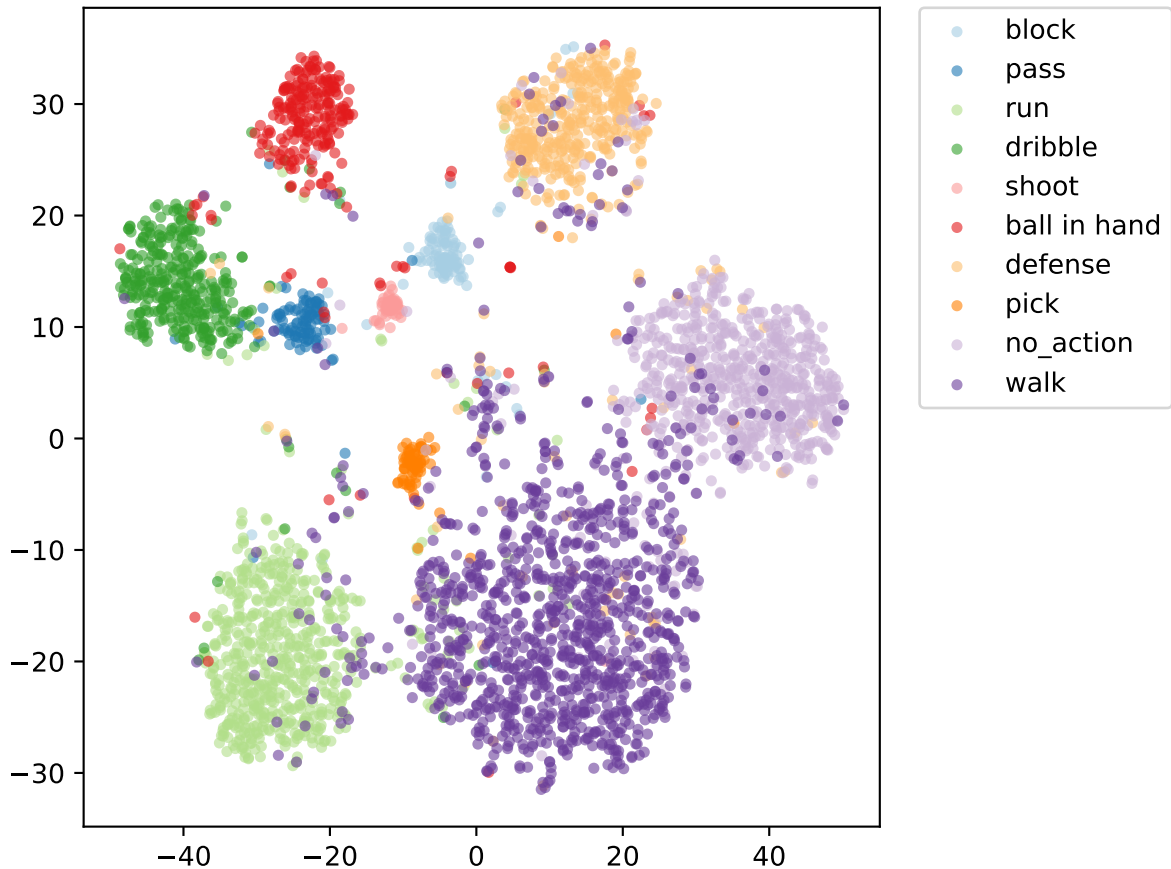

Supplement: Supplementary file 1 [file Data_Sheet_1.ZIP › figures/tsne_SpaceJam_after_training.pdf]

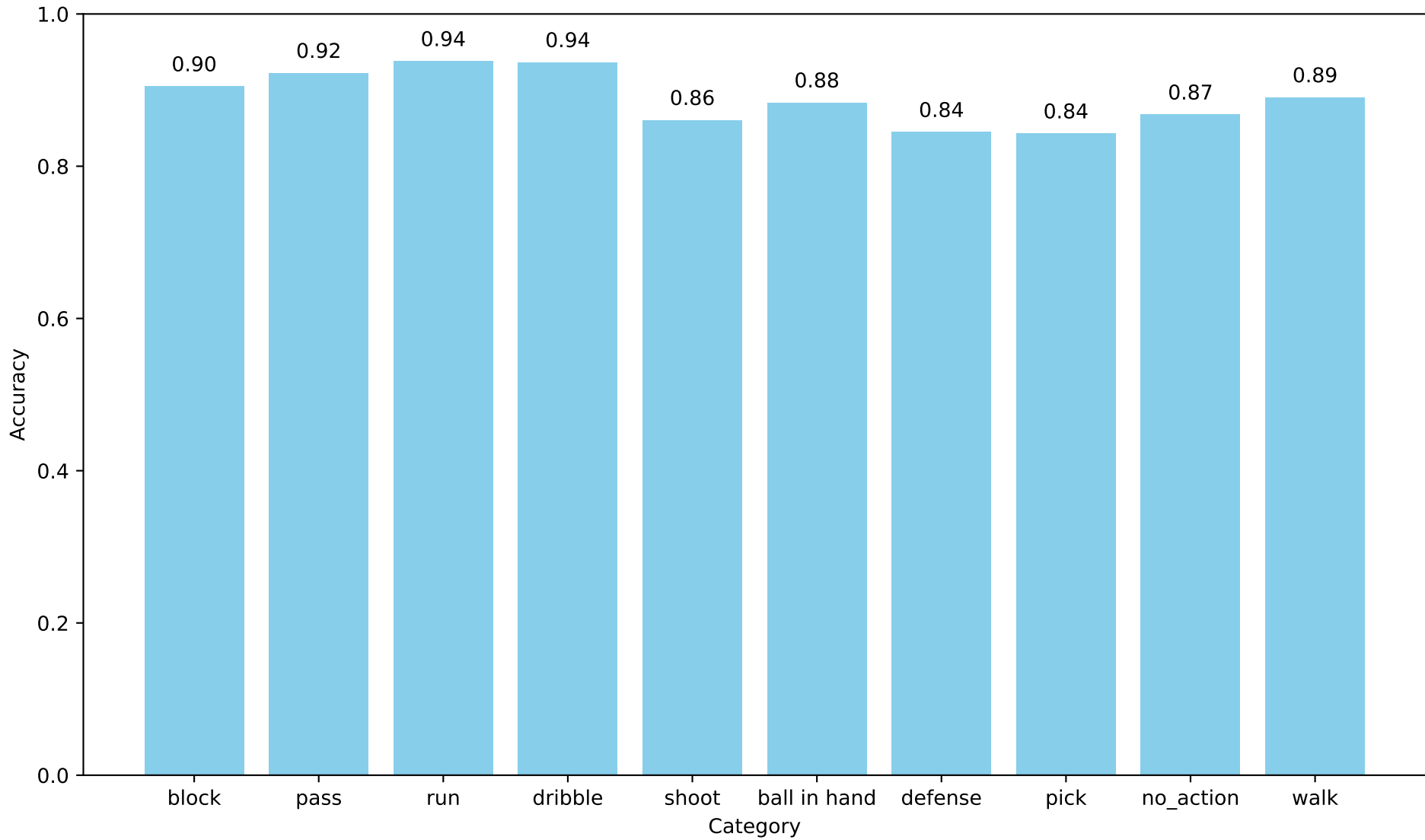

Supplement: Supplementary file 1 [file Data_Sheet_1.ZIP › figures/SpaceJam_category_accuracy.pdf]

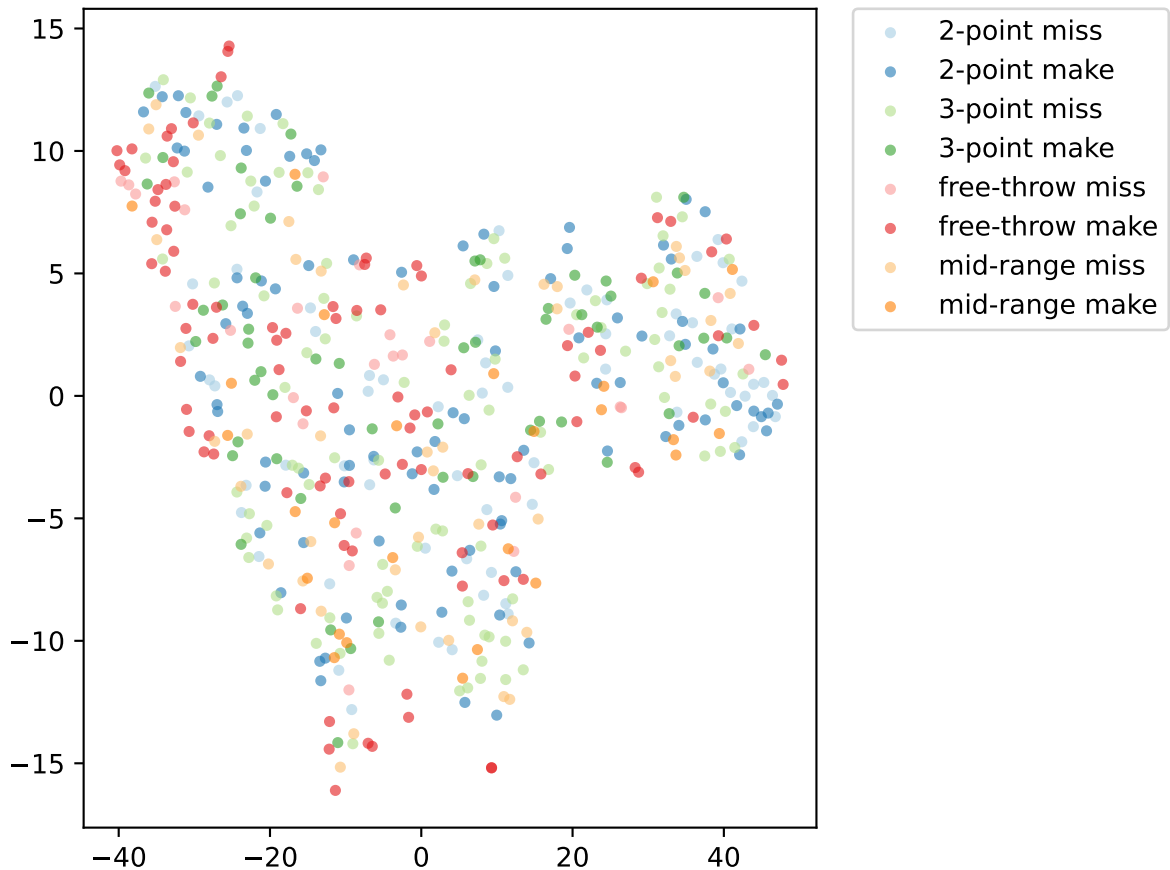

Supplement: Supplementary file 1 [file Data_Sheet_1.ZIP › figures/tsne_B51_before_training.pdf]

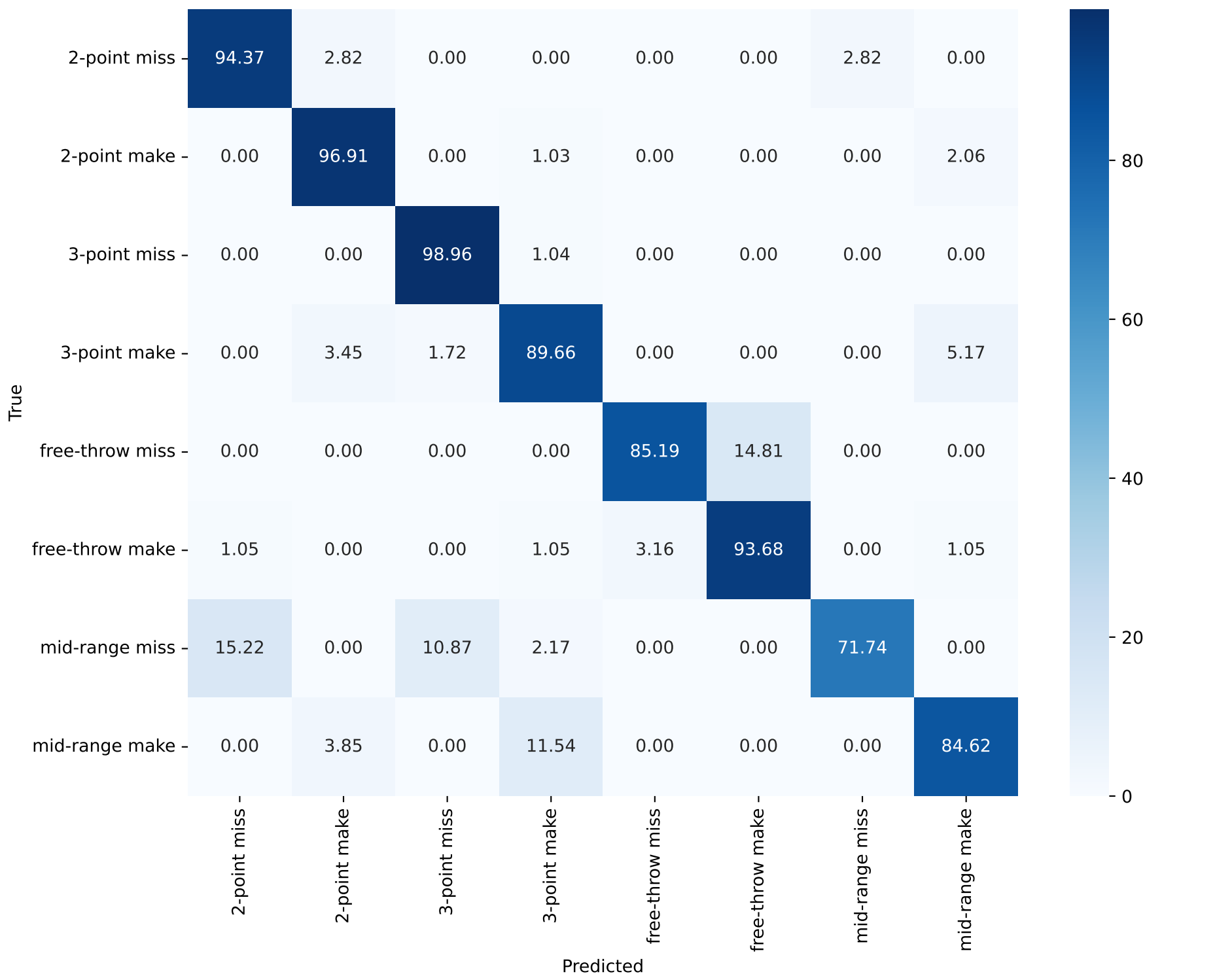

Supplement: Supplementary file 1 [file Data_Sheet_1.ZIP › figures/Basketball-51_confusion.pdf]

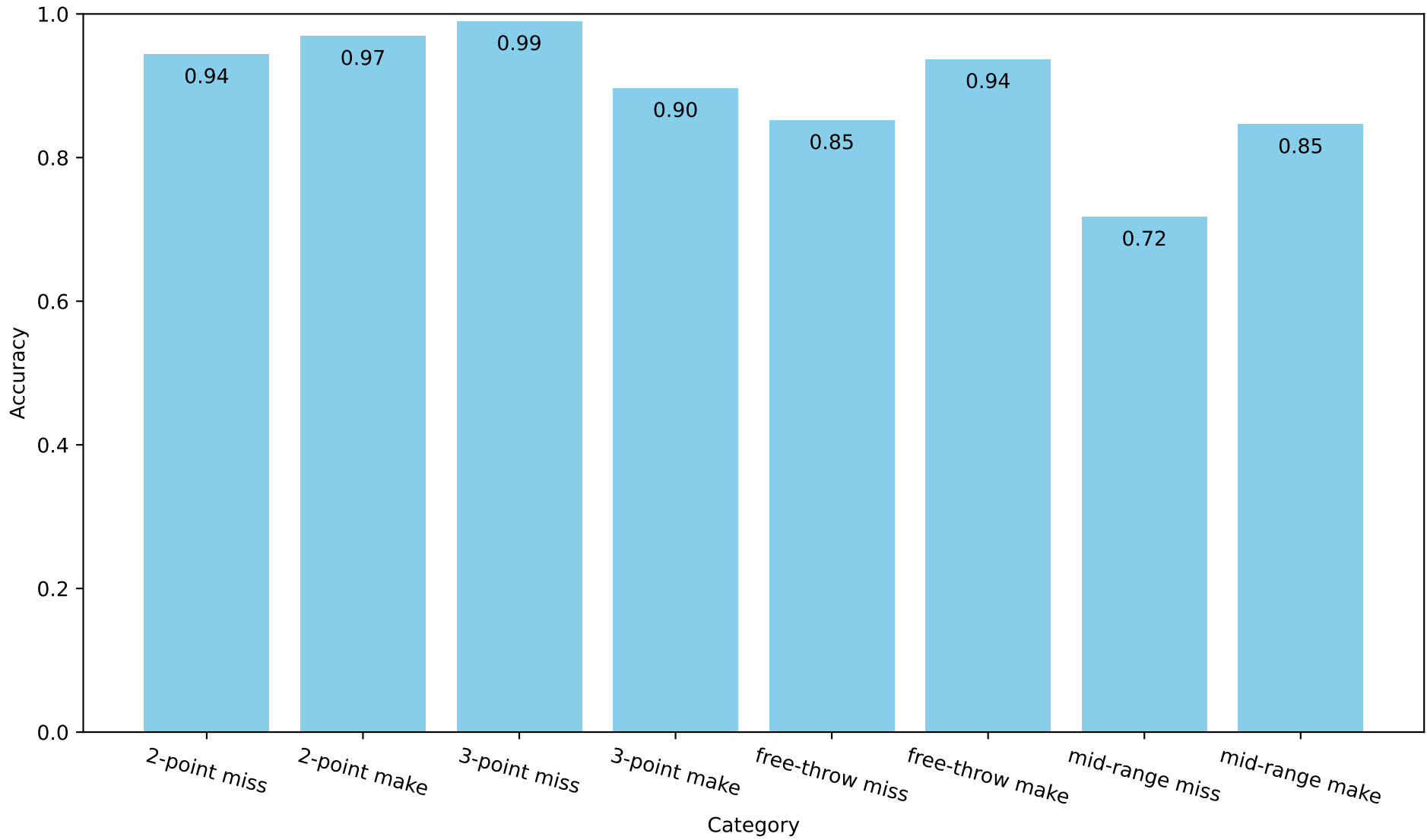

Supplement: Supplementary file 1 [file Data_Sheet_1.ZIP › figures/Basketball-51_category_accuracy.pdf]

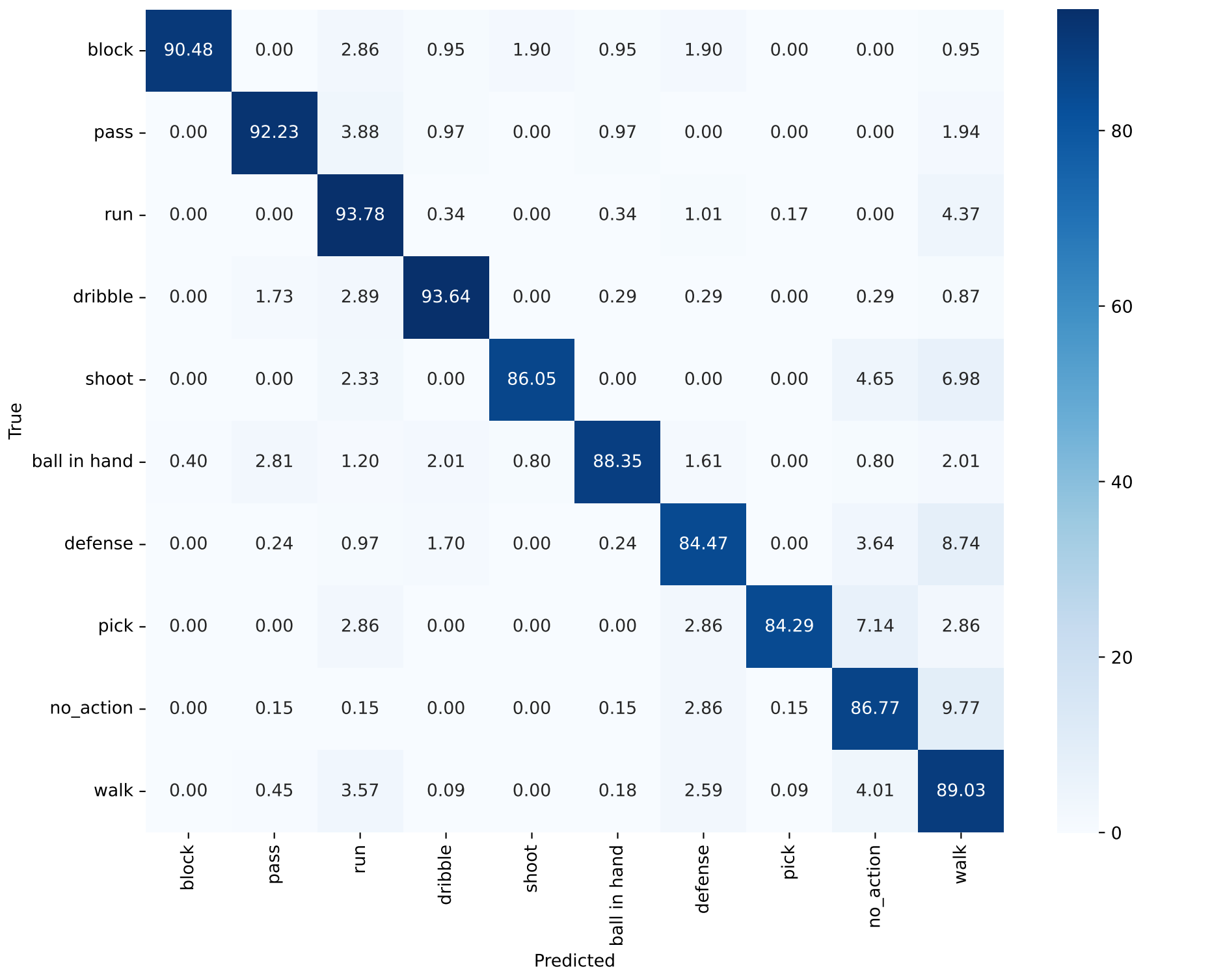

Supplement: Supplementary file 1 [file Data_Sheet_1.ZIP › figures/SpaceJam_confusion.pdf]

Number of samples

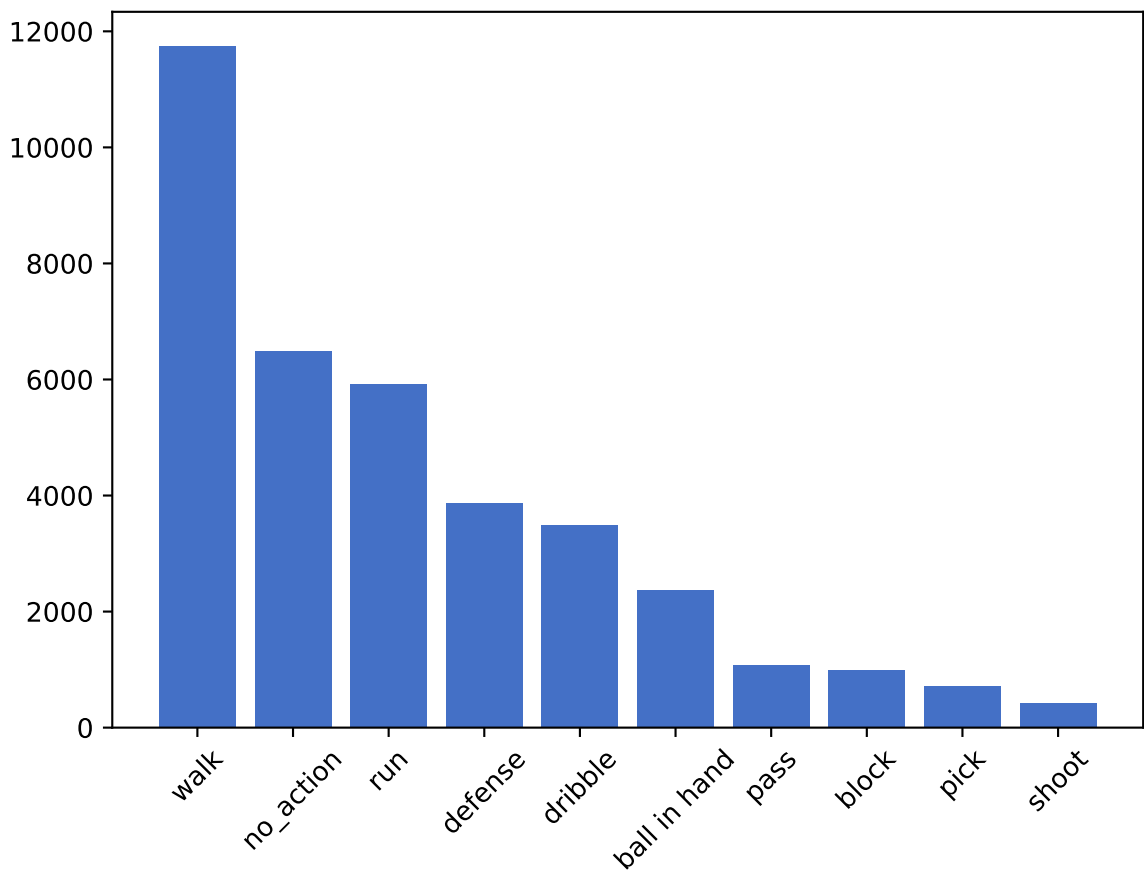

Supplement: Supplementary file 1 [file Data_Sheet_1.ZIP › figures/SpaceJam.pdf]

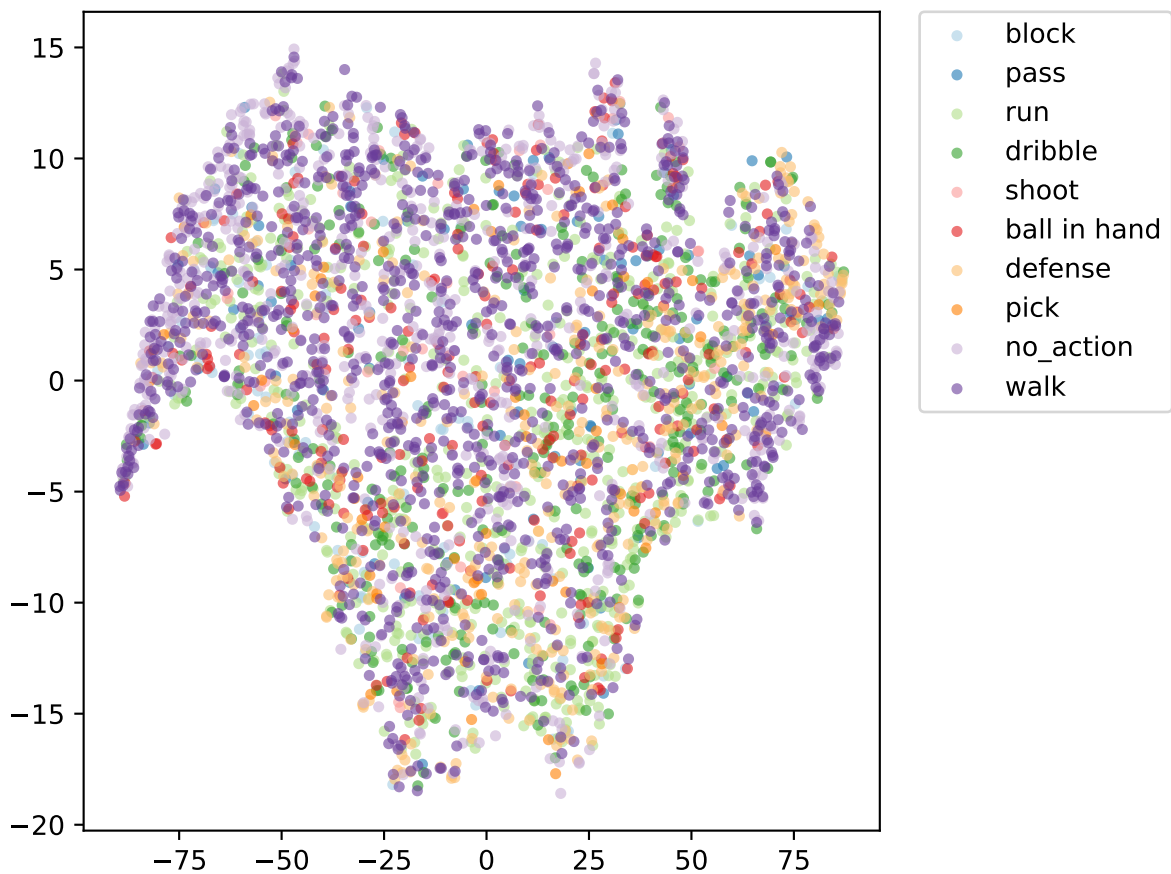

Supplement: Supplementary file 1 [file Data_Sheet_1.ZIP › figures/tsne_SpaceJam_before_training.pdf]
